# Modeling Isoprene Emission Response to Drought and Heatwaves Within MEGAN Using Evapotranspiration Data and by Coupling With the Community Land Model

**DOI:** 10.1029/2022MS003174

**Published:** 2022-12-21

**Authors:** Hui Wang, Xinchen Lu, Roger Seco, Trissevgeni Stavrakou, Thomas Karl, Xiaoyan Jiang, Lianhong Gu, Alex B. Guenther

**Affiliations:** ^1^ Department of Earth System Science University of California Irvine Irvine CA USA; ^2^ Department of Environmental Science, Policy and Management University of California Berkeley Berkeley CA USA; ^3^ Institute of Environmental Assessment and Water Research (IDAEA‐CSIC) Barcelona Spain; ^4^ Royal Belgian Royal Institute for Space Aeronomy (BIRA‐IASB) Brussels Belgium; ^5^ Department of Atmospheric and Cryospheric Sciences University of Innsbruck Innsbruck Austria; ^6^ Environmental Sciences Division Oak Ridge National Laboratory Climate Change Science Institute Oak Ridge TN USA

**Keywords:** isoprene, drought, MEGAN, evapotranspiration

## Abstract

We introduce two new drought stress algorithms designed to simulate isoprene emission with the Model of Emissions of Gases and Aerosols from Nature (MEGAN) model. The two approaches include the representation of the impact of drought on isoprene emission with a simple empirical approach for offline MEGAN applications and a more process‐based approach for online MEGAN in Community Land Model (CLM) simulations. The two versions differ in their implementation of leaf‐temperature impacts of mild drought. For the online version of MEGAN that is coupled to CLM, the impact of drought on leaf temperature is simulated directly and the calculated leaf temperature is considered for the estimation of isoprene emission. For the offline version, we apply an empirical algorithm derived from whole‐canopy flux measurements for simulating the impact of drought ranging from mild to severe stage. In addition, the offline approach adopts the ratio (*f*_PET_) of actual evapotranspiration to potential evapotranspiration to quantify the severity of drought instead of using soil moisture. We applied the two algorithms in the CLM‐CAM‐chem (the Community Atmosphere Model with Chemistry) model to simulate the impact of drought on isoprene emission and found that drought can decrease isoprene emission globally by 11% in 2012. We further compared the formaldehyde (HCHO) vertical column density simulated by CAM‐chem to satellite HCHO observations. We found that the proposed drought algorithm can improve the match with the HCHO observations during droughts, but the performance of the drought algorithm is limited by the capacity of the model to capture the severity of drought.

## Key Points

- New drought stress algorithms improve capability of Model of Emissions of Gases and Aerosols from Nature isoprene emission estimates
- Drought algorithms can improve the match with the HCHO observations during droughts
- The performance of the drought algorithm is limited by the capacity of the model to capture the severity of drought

## 1 Introduction

Isoprene plays a significant role in tropospheric chemistry due to the large amount of emission and its high chemical reactivity. It is the major species of Biogenic Volatile Organic Compounds (BVOCs) emitted from terrestrial vegetation and accounts for half of global BVOC emission (Guenther et al., 2012). Isoprene is an important precursor of ozone and secondary organic aerosol (SOA) (Claeys et al., 2004; Sillman, 1999), so accurately estimating isoprene emission is required to understand the ozone and SOA relevant chemical and physical processes for improving air quality and managing their climatic impact.

The emission of isoprene is affected by multiple environmental factors like light condition and temperature (Arneth et al., 2007; Guenther et al., 1993, 2006; Seco et al., 2020). Extreme weather events such as drought and heat waves can also play a role in determining isoprene emission (Ferracci et al., 2020; Potosnak et al., 2014; Seco et al., 2015). The isoprene biosynthesis and emission present a relatively higher drought tolerance than photosynthesis (Brilli et al., 2007; Brüggemann & Schnitzler, 2002; Fortunati et al., 2008; Sharkey & Loreto, 1993; Tingey et al., 1981), and isoprene emission decreases only under a severe drought situation because of the inhibition of substrate supply (Brilli et al., 2007; Fang et al., 1996; Pegoraro et al., 2004). Potosnak et al. (2014) hypothesized that isoprene emission could also be increased at the mild stage of drought indirectly by the increase of leaf temperature induced by reducing stomatal conductance. Supporting evidence of increased isoprene emissions under the mild stage of drought or heat stress has come from a number of studies in different environments (Kaser et al., 2022; Otu‐Larbi et al., 2020; Seco et al., 2015). Some of these studies (Emmerson et al., 2019; Jiang et al., 2018; Otu‐Larbi et al., 2020; Wang et al., 2021) have proposed algorithms to represent the influence of drought and heat stress on isoprene emission in earth system models. Niinemets (2010) established a conceptual model for describing the impact of drought and heatwave stress based on the severities of drought and heatwave. In this study, we adopt the model framework of Potosnak et al. (2014) to conceptualize the integrated impact of drought and heatwave. This approach simulates an isoprene emission rate that does not change with mild drought but is increased under moderate drought conditions as leaf temperature increases due to changes in stomatal conductance (Otu‐Larbi et al., 2020; Potosnak et al., 2014), which is considered as an indirect impact of drought on isoprene emission through changing leaf temperature. In severe drought events, isoprene emission drops because the substrate supply is eventually affected by the drought (Fortunati et al., 2008; Niinemets, 2010; Pegoraro et al., 2004; Potosnak et al., 2014), and we define this process as the direct impact of drought.

We introduce two drought stress algorithms in the Model of Emissions of Gases and Aerosols from Nature (MEGAN) v3.2 model in this study. We based our algorithm parameter coefficients on canopy scale flux measurements and scaled up the algorithms in regional and global models. The two drought stress algorithms adopted different ways to represent the impact of mild and moderate droughts on isoprene by stimulating leaf temperature. One major improvement of our drought algorithms is that we considered the two mechanisms of drought impact mentioned above on isoprene emission. Another major improvement of the offline algorithm is the use of a new drought indicator based on the ratio of actual evapotranspiration (ET) to potential evapotranspiration (PET) to evaluate the impact of water stress on isoprene emission. For model validation, we used satellite formaldehyde (HCHO) vertical column density to determine if the drought algorithms could improve the performance of HCHO simulation. In Section 2, we introduce the data sets, including in‐situ and satellite‐based data sets, that were used in this study. In Section 3, we introduce the emission model and the drought algorithms. In Section 4, the results of in‐situ and global simulations are presented and discussed.

## 2 Data Sets

### 2.1 Field Measurements

The isoprene flux measurements (Seco et al., 2015) used to parameterize the drought response algorithm were made at the Missouri Ozarks Forest AmeriFlux site (MOFLUX, 38.74°N, −92.2°W) (Gu et al., 2016) in 2012. The site is in the Baskett Wildlife Research and Education Center of the University of Missouri. The isoprene flux and meteorological variables were measured on a 32‐m scaffold tower, which is about 10m above the canopy. The site is covered by deciduous broadleaf forest with dominant tree species including white oak and black oak, shagbark hickory, sugar maple and eastern red cedar, and the dominant soils at the site are Weller silt loam and Clinkenbeard very flaggy clay loam (Gu et al., 2016). The campaign started on May 2 and ended on October 22 in 2012, during which a severe drought occurred. The campaign covered the whole growing season and the entire drought event and the associated variability of isoprene flux under the impact of drought, which enabled the development of a canopy scale drought stress algorithm for isoprene emission. More details about the campaign and measurements can be found in Seco et al. (2015).

### 2.2 Satellite Observations

Satellite HCHO observations were used to assess the drought algorithm for isoprene emission. Isoprene is the major source of HCHO in most rural regions (Palmer et al., 2003; Wolfe et al., 2016), and the satellite‐derived HCHO vertical column density has been widely used to investigate the variability of isoprene emission (Duncan et al., 2009; Stavrakou et al., 2018; Zheng et al., 2017; Zhu et al., 2017) and to constrain isoprene emission (Kaiser et al., 2018; Palmer et al., 2003; Stavrakou et al., 2009, 2015). We used the monthly HCHO vertical column density derived from the Ozone Monitoring Instrument (OMI) sensor (De Smedt et al., 2015) to investigate the impact of drought on isoprene emission and determine if updating the drought algorithm could improve the simulation of HCHO concentration distributions. The monthly Level‐3 HCHO vertical column density with 0.25° spatial resolution used in this study is from the website of the Royal Belgian Institute for Space Aeronomy (BIRA‐IASB, https://h2co.aeronomie.be) (De Smedt et al., 2012, 2015).

The satellite‐derived soil moisture (SM) from the ESA‐CCI data set (Dorigo et al., 2017; Gruber et al., 2019) was also used in this study. The ESA‐CCI SM data set v5.2 used here is a combined product that merged the SM derived from the passive and active microwave‐based sensors (Gruber et al., 2019). The ESA‐CCI SM data set v5.2 has a 0.25° spatial resolution with daily temporal frequency, and it was interpolated to the Community Land Model (CLM) model grids and compared to the surface SM simulated by CLM to evaluate the performance of the model.

In addition, a satellite‐based drought index, the evaporative stress index (ESI) (Anderson et al., 2011, 2013), was also used to upscale the offline drought algorithm. ESI is based on the ratio of actual ET to PET. ESI is derived from the remote sensing Atmosphere‐Land Exchange Inverse (ALEXI) model and satellite imagery of the thermal infrared band collected by the Geostationary Environmental Satellites. We downloaded the ESI index over 4‐week and 12‐week periods from the website of SERVIR GLOBAL (http://catalogue.servirglobal.net/Product?product_id=198).

## 3 Description of the Models

The MEGAN (Guenther et al., 2006, 2012) is a widely used flexible model framework for estimating BVOC emissions from individual sites (e.g., Seco et al., 2015, 2017) to the global scale (e.g., Chen et al., 2018; Müller et al., 2008; Opacka et al., 2021). MEGAN v3.2 calculates canopy scale flux of isoprene is estimated as:

(1) $$\begin{array}{c} F=\varepsilon LAI\gamma_{P}\gamma_{T}\gamma_{A}\gamma_{SM}\gamma_{C} \end{array}$$

where *F* (mg m^−2^ h^−1^), *ε* (mg m^−2^ h^−1^), and LAI (m^2^ m^−2^) represent the isoprene flux amount, the standardized emission factor, and the leaf area index, respectively. *γ*_P_, *γ*_T_, *γ*_A_, *γ*_SM_, and *γ*_C_ represent the activity factors for light, temperature, leaf age, drought, and CO_2_ inhibition, respectively.

The applications of MEGAN to estimate BVOC emission for chemistry transport models and earth system models use two approaches: an online version that couples MEGAN into a land ecosystem model (e.g., CLM) that can simulate the stomatal and leaf temperature change implicitly and an offline version that uses an independent MEGAN code. Therefore, we provide two different schemes for models with different complexity: an online isoprene response to drought scheme was directly implemented into a land ecosystem model using CLM as an example with an explicit temperature stimulation algorithm, and an empirical algorithm with a parameterized temperature stimulation algorithm was designed for the independent MEGAN code (Table 1).

**Table 1:** Descriptions of the Online Explicit Drought Stress (EDS) Algorithm and the Offline Parameterized Drought Stress (PDS) Algorithm

	The explicit drought stress (EDS) algorithm	The parameterized drought stress (PDS) algorithm
Drought Indicator	The water stress function (*β*_*t*_) in CLM 5	The ratio (*f*_PET_) of evapotranspiration (ET) to potential evapotranspiration (PET)
Mild or moderate drought impact induced by the leaf temperature change	The leaf temperature change induced by drought can be simulated directly by CLM. The change of leaf temperature could increase the isoprene emission following the temperature response curve in MEGAN (Guenther et al., 2012)	The impact of the leaf temperature change induced by drought is parameterized based on the canopy level flux measurements. The isoprene emission is increased during the drought following Equation 11
Severe drought impact induced by the biochemical substrate supply	When droughts get severe (*β*_*t*_ < 0.6), the drought impact would be modeled by using the maximum rate of carboxylation by the photosynthesis enzyme Rubisco (Vcmax) in CLM 5 following Equation 8 in this study	The limitation of the biochemical substrate supply induced by severe drought is parameterized based on the canopy level flux measurements, and it could decrease the isoprene emission when the drought gets severe following Equation 12

In this study, we simulated the isoprene flux at the MOFLUX site using both the online and the offline single‐point models. The online single‐point MEGAN was integrated into the single point version of CLM 5, SP‐CLM 5. The offline single‐point MEGAN is designed for site‐scale simulation and is written in Python. The single‐point simulations are driven by the meteorological measurements at the MOFLUX site. The SP‐CLM 5 adopted the framework of MEGAN v2.1 (Guenther et al., 2012) and used the canopy scale emission factor of 10 mg m^−2^ h^−1^ that represents the averaged emission potential of the whole canopy. The offline version MEGAN v3.2 used the leaf scale emission factor of 2.45 mg m^−2^ h^−1^, which represents the emission capacity of the unit leaf area.

We also conducted global scale simulations using the Community Atmosphere Model with Chemistry (CAM‐chem) model and used the results to evaluate the impact of drought on isoprene emission regionally and globally. The simulations were conducted on the NCAR Cheyenne HPE/SGI ICE XA System (CISL, 2019). The impact of the drought induced isoprene change on atmospheric chemistry was simulated by CAM‐chem. The gas‐chemistry and aerosol processes in CAM‐chem have been updated recently to better capture biogenic terpenoid (BVOC) relevant reactions and SOA formation (Emmons et al., 2020; Schwantes et al., 2020; Tilmes et al., 2019). In addition, since isoprene is the main contributor to formaldehyde in regions dominated by biogenic emissions (Palmer et al., 2003; Wolfe et al., 2016), we compared the model outputs of the HCHO vertical column density with the satellite product from OMI to study whether the model can capture the change of HCHO during the drought year.

### 3.1 Drought Indicators

Accurate estimation of drought severity is important for modeling the drought response of isoprene emission. Previous versions of MEGAN used SM as the indicator of drought (Bonn et al., 2019; Emmerson et al., 2019; Guenther et al., 2012; Otu‐Larbi et al., 2020; Potosnak et al., 2014; Seco et al., 2015), and a SM driven algorithm that required soil characteristics (wilting point) information as inputs. However, there are significant limitations with using SM as the drought indicator. First, it is challenging to assign the thresholds for defining drought severity for isoprene emission modeling. For instance, the wilting point, the SM at which a plant cannot extract water from soil, is used to define the severity of drought in MEGAN v2.1. However, some previous studies (Huang et al., 2015; Opacka et al., 2022; Potosnak et al., 2014; Seco et al., 2015) have shown that the wilting point estimates are a major source of uncertainty for isoprene emission estimation during the drought. Second, the SM driven drought algorithm is sensitive to the accuracy of the SM inputs, and the systematic errors of the SM data sets from land surface models or satellites will directly affect the estimation of isoprene emission (Emmerson et al., 2019; Opacka et al., 2022). In addition, different SM data sets will also affect the performance of algorithms. For instance, SM estimated by various models or observational systems (e.g., satellite or in‐situ measurements) could represent different soil depths, so different empirical thresholds are required to simulate the same impact on isoprene emission (Opacka et al., 2022). Third, the hydrologic stress of an ecosystem is affected not only by soil water availability (SWA) but also the atmospheric vapor pressure deficit (VPD), which represents the atmospheric demand for water (Novick et al., 2016; Park Williams et al., 2013; Porporato et al., 2001; Schulze, 1986).

In this study, we introduce direct vegetation water stress indicators for evaluating the impact of drought on isoprene emission. In the online version of MEGAN in CLM 5, we adopted the water stress function (*β*_*t*_) to drive the isoprene response to water stress. The *β*_*t*_ is a water stress indicator, ranging between 0 and 1 in CLM, and is calculated as:

(2) $$\beta_{t}=\sum_{i=1}^{n}w_{i}r_{i}$$

where *w*_i_ and *r*_i_ represent the wilting factor and the fraction of root distribution for different plant functional types (PFT) in soil layer *i* with *n* layers in total. The wilting factor in CLM 4.5/5 is as (Oleson et al., 2013):

(3) $$\begin{array}{c} w=\frac{\psi_{c}-\psi }{\psi_{c}-\psi_{o}}\cdot \left(\frac{\theta_{s}-\theta_{ice}}{\theta_{s}}\right) \end{array},$$

where *ψ* is the soil matric potential (mm), *ψ*_*c*_ and *ψ*_*o*_ are the soil water potential (mm) when stomata are fully closed and fully open, respectively. *ψ*_*c*_ and *ψ*_*o*_ are PFT‐dependent parameters, and *θ*_ice_ is the volumetric soil ice content (m^3^ m^−3^). More details about the calculation of *β*_*t*_ can be found in Oleson et al. (2013). The wilting factor is different from the wilting point. The wilting point is an absolute value based on the soil texture only (Chen & Dudhia, 2001), while the wilting factor is a relative variable to describe the severity of ecosystem water stress based on soil wetness and PFT types. The wilting factor considers the openness of the stomata, which connects plant water stress with soil wetness.

For the offline version model, we used the ratio (*f*_PET_) of actual ET to PET to indicate drought. Compared to previous studies using SM as the proxy of drought severity (Bonn et al., 2019; Otu‐Larbi et al., 2020; Wang et al., 2021), the ET‐based drought indicator is expected to provide a more direct measure of water stress on vegetation (Yan et al., 2015).

The half‐hour ET (mm day^−1^) is calculated as:

(4) $$\begin{array}{c} ET=\frac{LE}{\lambda } \end{array}$$

where LE is the latent heat flux (MJ m^−2^ day^−1^) and λ (MJ kg^−1^) is the latent heat of vaporization calculated as (Stull, 1988):

(5) $$\begin{array}{c} \lambda =2.501-0.00237\cdot T \end{array}$$

where *T* is the air temperature (°C). We treated the reference ET as PET, and calculated it using the Penman–Monteith equation as:

(6) $$PET=\frac{0.408∆\left(R_{n}-G\right)+\gamma \frac{\;37}{T+273.15}u_{2}\left(e_{s}-e_{a}\right)}{∆+\gamma \left(1+0.34u_{2}\right)}$$

In Equation 6, *R*_*n*_ is the net radiation (MJ m^−2^ day^−1^), *G* is the soil heat flux density (MJ m^−2^ day^−1^), Δ (kPa °C^−1^) is the slope of the saturation water vapor pressure at air temperature *T* (°C), *γ* is the psychrometric constant (kPa °C^−1^) and *u*_2_ is the wind speed at 2m height (m s^−1^). *e*_*s*_ and *e*_*a*_ denote the saturation vapor pressure (kPa) at air temperature *T* and the actual vapor pressure (kPa), respectively. To develop the algorithm, we only take the values of *f*_PET_ on relatively sunny days when the incoming shortwave radiation is above 500 W m^−2^. We filled the missing values with the mean of the remaining data points in that day, then we conducted a 7‐day smoothing to the *f*_PET_. We also adopted a *f*_PET_ based satellite drought index, ESI (Anderson et al., 2011, 2013), to spatially upscale the algorithm.

### 3.2 Online Explicit Drought Stress (EDS) Algorithm

The online drought stress algorithm introduced here is coupled to the CLM model and is referred to here as the Explicit Drought Stress (EDS) algorithm. As we mentioned above, there are two main mechanisms driving the drought impact on isoprene emission: (a) the indirect impact of drought through changing leaf temperature which drives enzymatic activity, and (b) the direct impact of drought by affecting substrate supply. CLM is a process‐based model with comprehensive considerations of the plant physiology, therefore, it can provide inputs for directly simulating the drought impact of leaf temperature and substrate availability. Currently, there are two stomatal conductance models available in CLM 5. Our work is based on the Ball‐Berry conductance model described in Collatz et al. (1991) and Sellers et al. (1996), and the leaf stomatal conductance (*g*_*s*_, μmol s^−1^ m^−2^) as described by Collatz et al. (1991):

(7) $$\begin{array}{c} g_{s}=m\frac{A_{n}}{C_{s}/P_{atm}}h_{s}+b\beta_{t} \end{array}$$

where *m* is a PFT‐dependent parameter, *A*_*n*_ is the leaf net photosynthesis (μmol m^−2^ s^−1^), *C*_*s*_ is the partial pressure of CO_2_ at the leaf surface (Pa), P_atm_ is the atmospheric pressure (Pa), and *h*_*s*_ is the leaf surface humidity at the leaf surface, *b* is the minimum stomatal conductance (μmol s^−1^ m^−2^) and *β*_*t*_ is the water stress function described in the previous section. *β*_*t*_ can decrease *g*_*s*_ in response to drought leading to an increase in leaf temperature.

The drought stress is represented by *γ*_SM_ in the MEGAN model as shown in Equation 1. The impact of the severe drought on isoprene emission is presented in Jiang et al. (2018). The drought algorithm in Jiang et al. (2018) is calculated as:

(8) $$\begin{array}{c} \left\{\begin{array}{c} \gamma_{SM}=1\;\left(\beta_{t}\geq 0.6\right)\\ \gamma_{SM}=V_{c\;\max}/\alpha \;\left(0<\beta_{t}<0.6\right)\\ \gamma_{SM}=0\left(\beta_{t}=0\right) \end{array}\right. \end{array}$$

where *γ*_SM_ is the isoprene emission activity factor response to drought, *β*_*t*_ is the water stress function, *V*_*c*max_ is the maximum rate of carboxylation by the photosynthesis enzyme Rubisco, and *α* (=37) is an empirical parameter derived from the observations at the MOFLUX site in 2012 (Seco et al., 2015). With this algorithm, the isoprene emission will not be affected when drought is in the mild and moderate stage (*β*_*t*_ ≥ 0.6). In the severe drought condition (*β*_*t*_ < 0.6), when photosynthesis and the supply of carbon substrates is limited, the emission of isoprene will be decreased. Since the impact of drought on leaf temperature can be simulated by CLM, the MEGAN model integrated within CLM uses CLM parameters to drive the two mechanisms that control how drought influences isoprene emission. However, since this online version of MEGAN relies on CLM to calculate parameters based on detailed biogeochemistry and plant physiology processes, it cannot be directly applied in simpler model frameworks. Therefore, we developed a parameterized drought algorithm for offline simulations as described in Section 3.3.

### 3.3 Offline Parameterized Drought Stress (PDS) Algorithm

In MEGAN v2 (Guenther et al., 2006), the drought (SM) impact on isoprene emission is described by a simple empirical algorithm that represents the isoprene emission activity factor response to drought, *γ*_SM_, as:

(9) $$\begin{array}{c} \left\{\begin{array}{c} \gamma_{SM}=1\left(\theta >\theta_{1}\right)\\ \gamma_{SM}=\left(\theta -\theta_{w}\right)/∆\theta_{1}\\ \gamma_{SM}=0\left(\theta <\theta_{w}\right) \end{array}\right.\;\left(\theta_{w}<\theta <\theta_{1}\right) \end{array}$$

where *θ* is SM, *θ*_*w*_ is wilting point. Δ*θ*_1_ is an empirical parameter and *θ*_1_ is defined as *θ*_*w*_ + Δ*θ*_1_. The initial version, MEGAN v2 (Guenther et al., 2006), assigned Δ*θ*_1_ a value of 0.06 m^−3^ m^−3^ based on the potted plant enclosure measurements of Pegoraro et al. (2004). The subsequent version, MEGAN v2.1 (Guenther et al., 2012), assigned a value of 0.04 m^−3^ m^−3^. As mentioned in Section 3.1, there are three main limitations of using SM to evaluate drought severity: uncertainty of thresholds, inconsistences of wilting point and SM values among different data sets and neglecting the impact of atmospheric VPD. In addition, the algorithm in MEGAN v2.1 cannot represent the indirect impact of drought on isoprene through increased leaf temperature induced by lower stomatal conductance.

Two aspects were considered for the new offline algorithm: (a) a reliable way to quantify the severity of drought impacts on inhibiting vegetation biochemical substrates and (b) consideration of the indirect impact of the drought on enhancing isoprene emission through elevated leaf temperature. We refer to this new offline drought algorithm as the Parameterized Drought Stress (PDS) algorithm with empirical coefficients derived from canopy scale observations of isoprene flux during 2012 at the MOFLUX site. The PDS approach calculates isoprene drought response as:

(10) $$\gamma_{sm}=\gamma_{sm\_max}\cdot \gamma_{sub}\cdot \gamma_{lt}$$

(11) $$\gamma_{sub}=\frac{1}{1+b_{1}\cdot e^{a_{1}\cdot \left(f_{pet}-0.2\right)}}$$

(12) $$\gamma_{lt}=\frac{1}{\gamma_{sm\_max}}+\frac{\left(1-\frac{1}{\gamma_{sm\_\max}}\right)}{1+b_{2}\cdot e^{a_{2}\cdot \left(1.3-f_{pet}\right)}}$$

**Figure 1:**
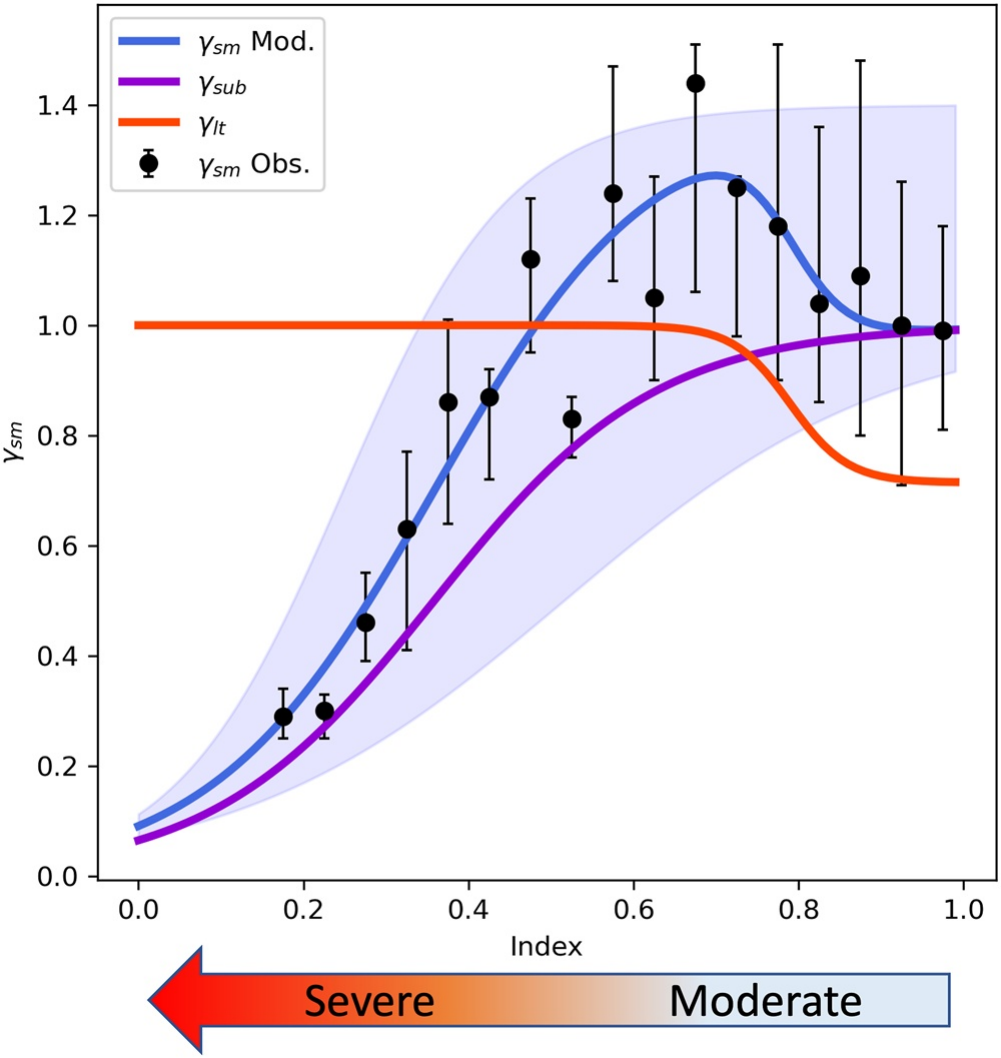
The parameterized drought stress (PDS) algorithm for offline MEGAN simulations. *γ*_sm_ represents the response of isoprene emission to drought. The index, 7 days running averaged *f*_PET_, represents the severity of drought. The observed values were grouped into 0.05 index intervals, and the upper (lower) cap of error bars represents the upper (lower) quartile. The blue, red and purple solid lines represent the fitting model of *γ*_sm,_ *γ*_lt_, and *γ*_sub,_ respectively, and the blue shadow represents 95% confident intervals of the fitting model of *γ*_sm_.

In this algorithm, *γ*_sm_max_ (=1.4) represents the maximum value of *γ*_sm_. *γ*_sub_, and *γ*_lt_ account for the impacts of the substrate supply (sub) and the leaf temperature (lt) stimulation, respectively. The parameters *a*_1_ (=−7.45), *a*_2_ (=−28.76), *b*_1_ (=3.26), and *b*_2_ (=2.35 × 10^6^) control the shape of the curve. We propose *f*_PET_ as a suitable drought indicator because the transpiration of plants would decrease with stomata closure when plants feel the water stress (Hanson, 1991). We normalized the 7‐day running averaged *f*_PET_ for the 2012 MOFLUX study to span the range of 0–1 by using the minimum value of 0 and the maximum value of 0.82 (95% percentile of the 7‐day running averaged *f*_PET_ between 2006 and 2017). Other vegetation water stress indexes or indicators (e.g., ESI drought index in this study) can alternatively be used as inputs after being normalized the maximum to 1 and the minimum to 0, and this feature is important for using the PDS algorithm in other model framework with other drought indexes or inputs. The PDS algorithm responses of *γ*_sm_, *γ*_sub_, and *γ*_lt_ to the normalized drought index are shown in Figure 1. The estimates of *γ*_sm_ derived from the ratios between the flux observations (*F*_obs_) and the offline model outputs (*F*_mod_) are calculated as:

(13) $$\begin{array}{c} \gamma_{sm\_obs}=\frac{F_{obs}}{F_{\mathrm{mod}}} \end{array}$$

The normalized drought indexes were divided into bins with an interval of 0.05, and the averaged values of *γ*_sm_obs_ in each bin of normalized *f*_PET_ were used to fit the model. All averaged ratios were divided by the first value of the array to set *γ*_sm_obs_ to unity when there is no drought. *γ*_lt_ increases when drought gets into the moderate stage (index <0.9) and stays stable, while the *γ*_sub_ decreases when drought is severe enough (index <0.7) to affect the supply of substrate for isoprene synthase (Figure 1).

## 4 Results and Discussion

### 4.1 Comparison of Drought Indicators

**Figure 2:**
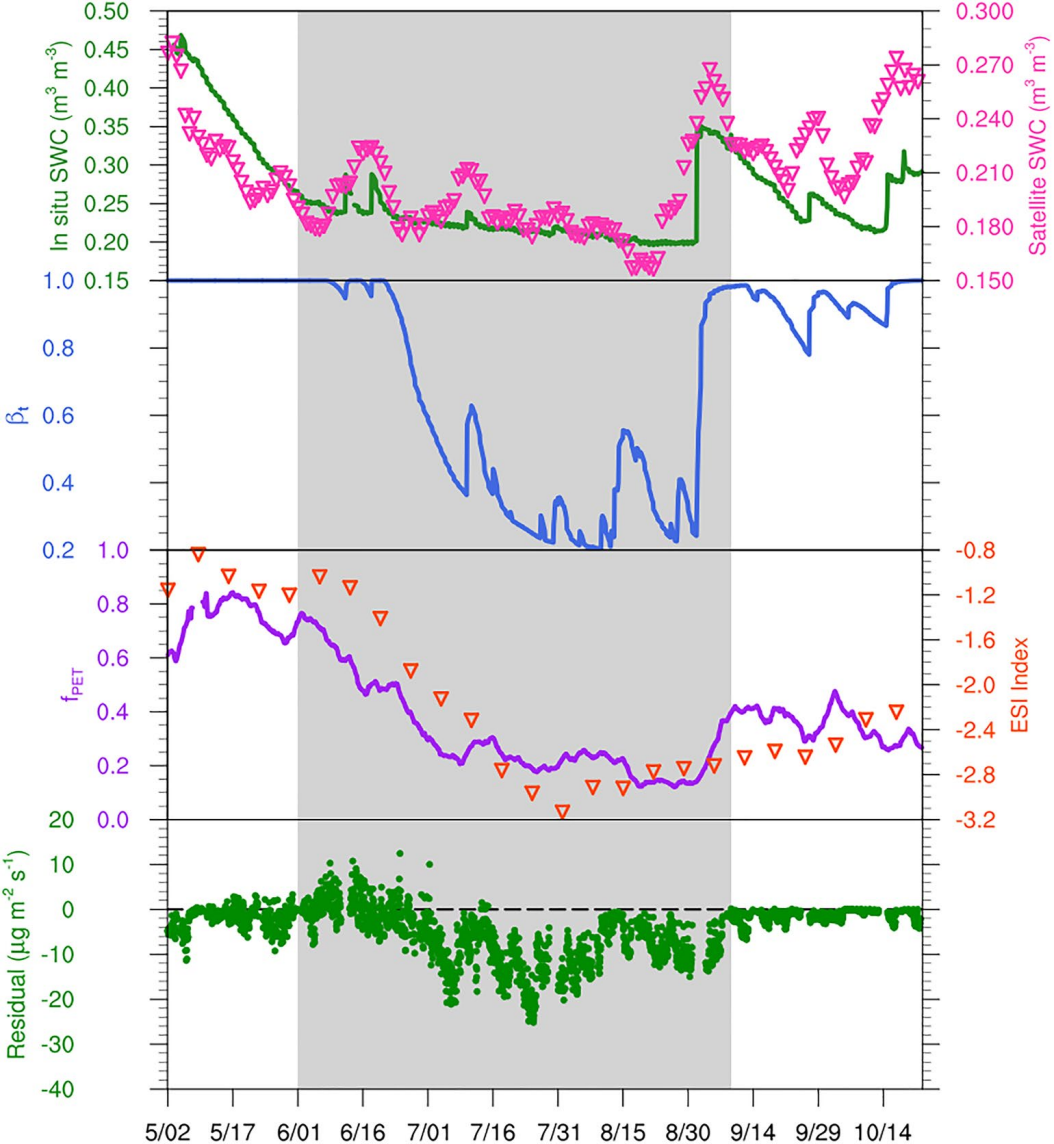
Comparison of drought indicators at the MOFLUX site in 2012. The drought period is indicated with a gray background. The first panel represents the 10 cm soil water content (SWC) from the in situ observations at the MOFLUX site (green solid line) and the surface soil water content (SWC) from the ESA‐CCI satellite product (pink triangles). The second panel represents the *β*_*t*_ simulated by the Community Land Model (blue solid line), and the third panel represents the drought indexes (*f*_PET_) based on the ratio of the real evapotranspiration (ET) and the potential evapotranspiration (PET) from the in situ observations (purple solid line) and the satellite 12‐week ESI drought index (orange triangles). The last panel represents the residuals of MEGAN v3.2 without the drought algorithm (green dots).

We compared the different drought indicators for the 2012 MOFLUX study as shown in Figure 2. We also calculated the residuals between the isoprene flux observed at the MOFLUX site and the isoprene flux modeled by the independent MEGAN v3.2 without the drought algorithm. The independent MEGANv3.2 was driven by the meteorological inputs observed at the site. The residuals (green dots) of the model increase at the beginning of the drought and decrease near the end of the drought (Figure 2). The SM data sets from the in‐situ measurements (green solid line) at 10 cm depth and the ESA‐CCI SM (Gruber et al., 2019) satellite product (pink triangles) show a similar pattern of SM that is not fully consistent with the changes of the model residual. The SM decreases before the drought starts to affect the emission of isoprene from 3 May to 2 June. We also presented the time‐series of other drought indicators for the MOFLUX site in 2012. The variabilities of *f*_PET_ and *β*_*t*_ are more consistent with the change of the model residuals (Figure 2). Using SM as a drought indicator is an indirect way to reflect the water stress of vegetation. The *f*_*P*ET_ and *β*_*t*_ used in the offline and online drought algorithms in this study are a more direct representation of water stress for vegetation. More importantly, using *f*_PET_ and *β*_*t*_ could relatively decrease the need to set wilting point threshold values, which are a major contributor to the uncertainties described in Section 3.1. We used the 2012 observations from the MOFLUX site as the benchmark to choose the suitable inputs for scaling up the model. We tested different normalized drought indexes using the PDS algorithm in this study, and the results are shown in Figure S1 in Supporting Information S1. The satellite‐based ESI drought index over a 12‐week period shows a good consistency with the *f*_PET_ behavior (Figure 2) and can therefore be used to scale up the PDS offline algorithm for regional to global scale modeling. Compared with the approach using SM as the drought indicator, the approaches using the direct plant water stress indexes is a more suitable way to simulate the impact of drought on isoprene emission by connecting plant physiology with the soil and atmosphere wetness. However, these indexes are also limited by the models and parameters used for estimating the severity of water stress. For instance, there are still some discrepancies among *f*_PET_, *β*_*t*_, and ESI (e.g., start date of the water stress) even though their general patterns are the same for the MOFLUX site in 2012.

### 4.2 Site Scale Simulations

**Figure 3:**
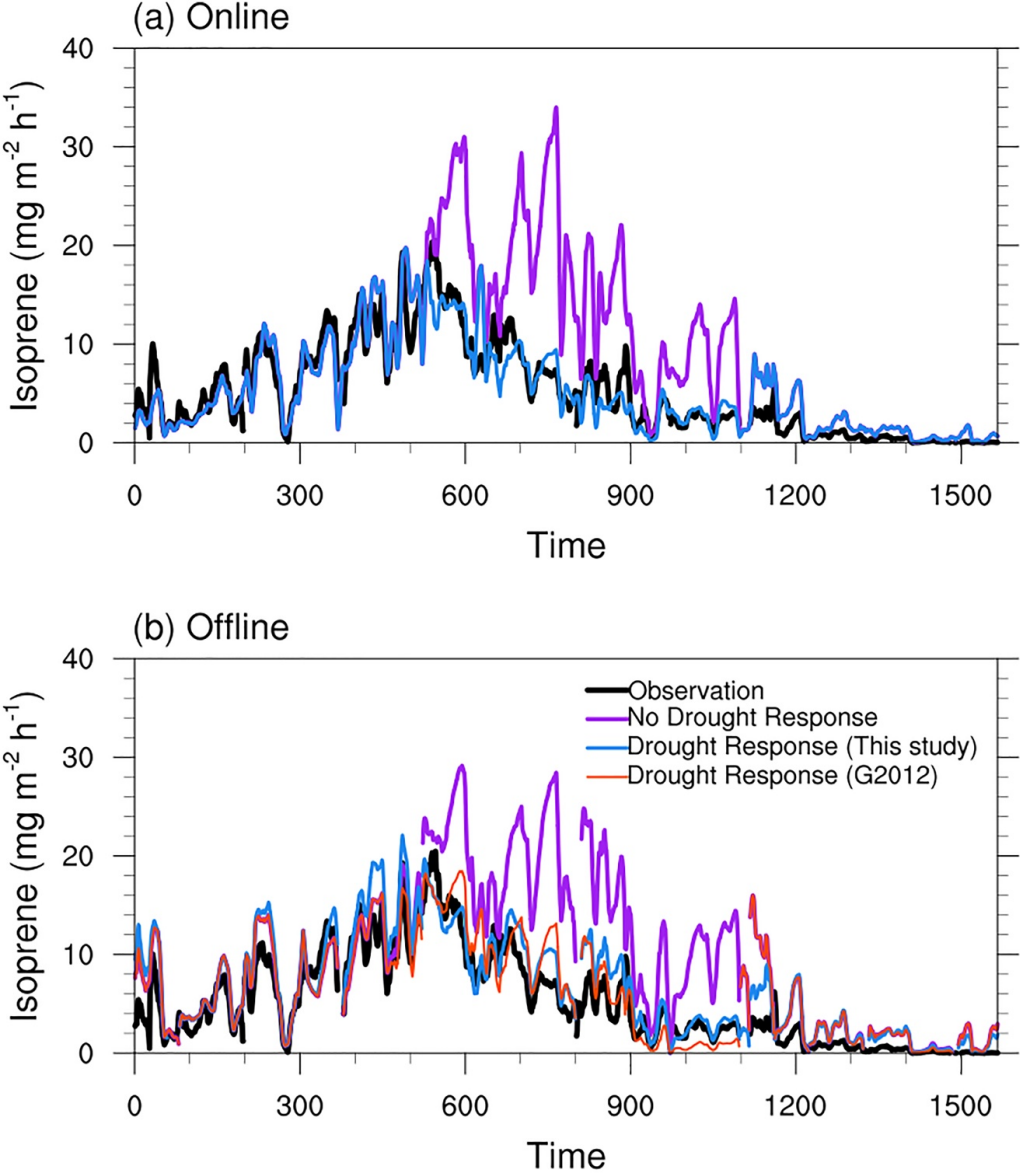
The 9‐hr running averaged time series of the hourly isoprene flux during the daytime observed and simulated by (a) SP‐CLM‐MEGAN and (b) the offline version MEGAN v3.2 at the MOFLUX site in 2012. The observations, model results with and without the drought algorithm in this study are presented by the black, blue and purple solid lines, respectively. The offline model results by the original drought algorithm are presented by the orange line in (b).

**Figure 4:**
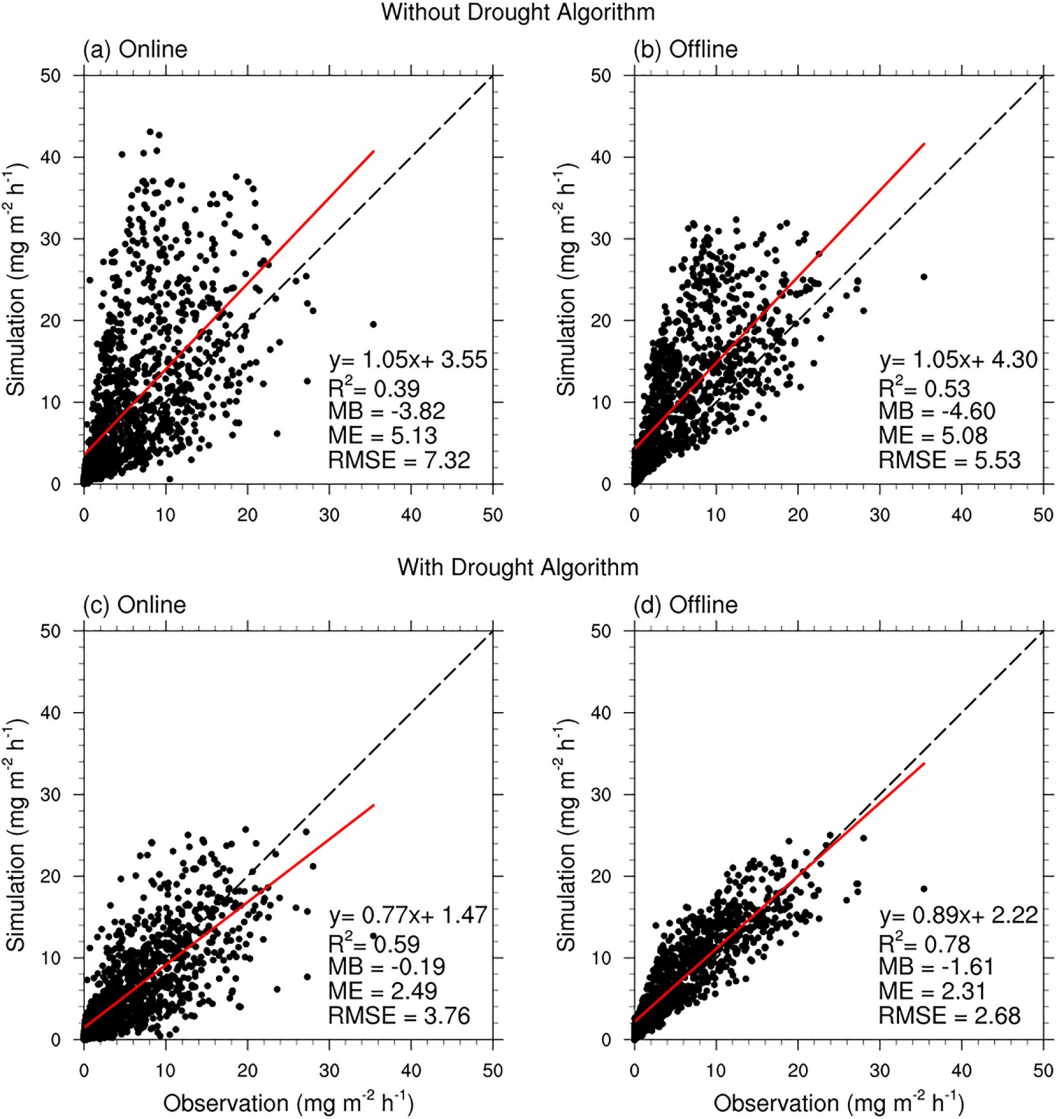
Scatter plots of measured and modeled hourly isoprene fluxes during the daytime. The results of SP‐CLM‐MEGAN with and without the EDS drought algorithm are presented in (a) and (c). The results of the offline version MEGAN v3.2 model with and without the PDS drought algorithm are presented in (b) and (d). MB, ME, and RMSE are abbreviations of the mean bias, the mean error and the root mean square error with the unit of mg m^−2^ h^−1^, respectively.

We ran site scale simulations to investigate the impact of the drought on isoprene emission at the MOFLUX site. The online model results came from SP‐CLM 5 model, and the offline model results from the independent version MEGAN v3.2. Both models were driven by the meteorology variables measured at the MOFLUX site in 2012. The input of the PDS algorithm for the offline model is the normalized 7‐day running average *f*_PET_ as shown in Figure 2. The comparisons between the model results and observations are shown in Figures 3 and 4. The online and offline models both overestimated the isoprene flux during the drought period when drought was neglected (Figure 3). When the drought algorithm was adopted, both the online and offline versions of the model captured the drought impact on isoprene emission (Figure 3). We also presented the results of the offline model embedded in the original drought algorithm (Equation 9) with *θ*_*w*_ of 0.194 (orange line in Figure 3). The original algorithm can have a comparable performance with the new one in this study with a suitable wilting point, however, the difficulty still exists for determining the wilting point. The difference between the drought algorithms developed in this study and the previous algorithms is discussed in Section 4.4.

The performance of the SP‐CLM model with embedded MEGAN and that of the offline MEGAN v3.2 model differed in the simulation of isoprene flux at MOFLUX in 2012 (Figure 4). This is at least partly because they have different canopy models for simulating the environmental conditions including leaf temperature and light conditions. Furthermore, both the online and offline models captured the variabilities of isoprene flux when the drought algorithms were adopted ((c) and (d) in Figure 4). For the SP‐CLM‐MEGAN model, the R^2^ increased from 0.39 to 0.59, and the mean bias (MB), the mean error (ME) and the root mean square error (RMSE) decreased from −3.82, 5.13, and 7.32 mg m^−2^ h^−1^ to −0.19, 2.49, and 3.76 mg m^−2^ h^−1^, respectively. For the offline version MEGAN v3.2, the *R* ^2^ increased from 0.53 to 0.78. The MB, the ME and the RMSE decreased from −4.6, 5.08, and 5.53 mg m^−2^ h^−1^ to −1.61, 2.31, and 2.68 mg m^−2^ h^−1^, respectively.

**Figure 5:**
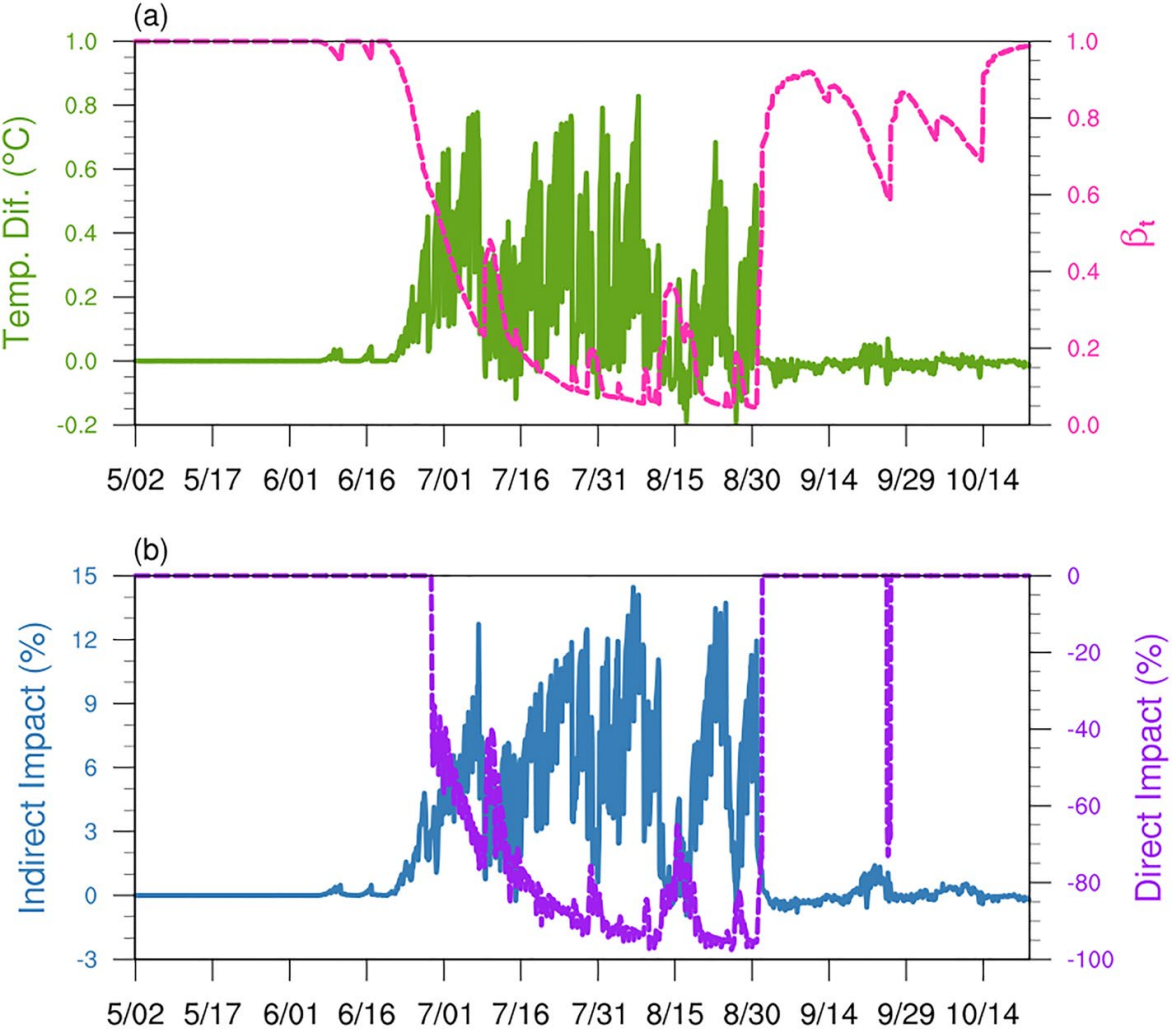
The change of leaf temperature and isoprene emission change simulated by SP‐CLM‐MEGAN during the drought at the MOFLUX site in 2012. The leaf temperature change induced by drought (green solid line) and *β*_*t*_ simulated by SP‐CLM (pink dashed line) are presented in (a), and the indirect impact of drought caused by stimulating temperature (blue solid line) and the direct impacts of drought caused by affecting substrate availability on isoprene emission (purple dashed line) are presented in (b).

We used the results of SP‐CLM‐MEGAN model to present (a) the indirect impact of drought through changing leaf temperature and (b) the direct impact of drought by affecting substrate supply on isoprene emissions at the MOFLUX site in 2012. As shown in Figure 5, the impact of drought on leaf temperature appears with the onset of water stress (*β*_*t*_ < 1). By decreasing the stomatal conductance following Equation 7, the leaf temperature could increase up to 0.83°C at the MOFLUX site during the 2012 drought ((a) in Figure 5). Correspondingly, the change of leaf temperature could lead to an increase of isoprene emission up to 14.5% ((b) in Figure 5). Meanwhile, the direct impact of drought on substrate supply started to affect the simulated isoprene emission when *β*_*t*_ is lower than 0.6 according to Equation 8. The direct impact of drought is related to the severity of drought in CLM, and the change of isoprene emission could be near 100% when the drought is very severe (Figure 5). The final impact of drought is the combination of these two mechanisms.

**Figure 6:**
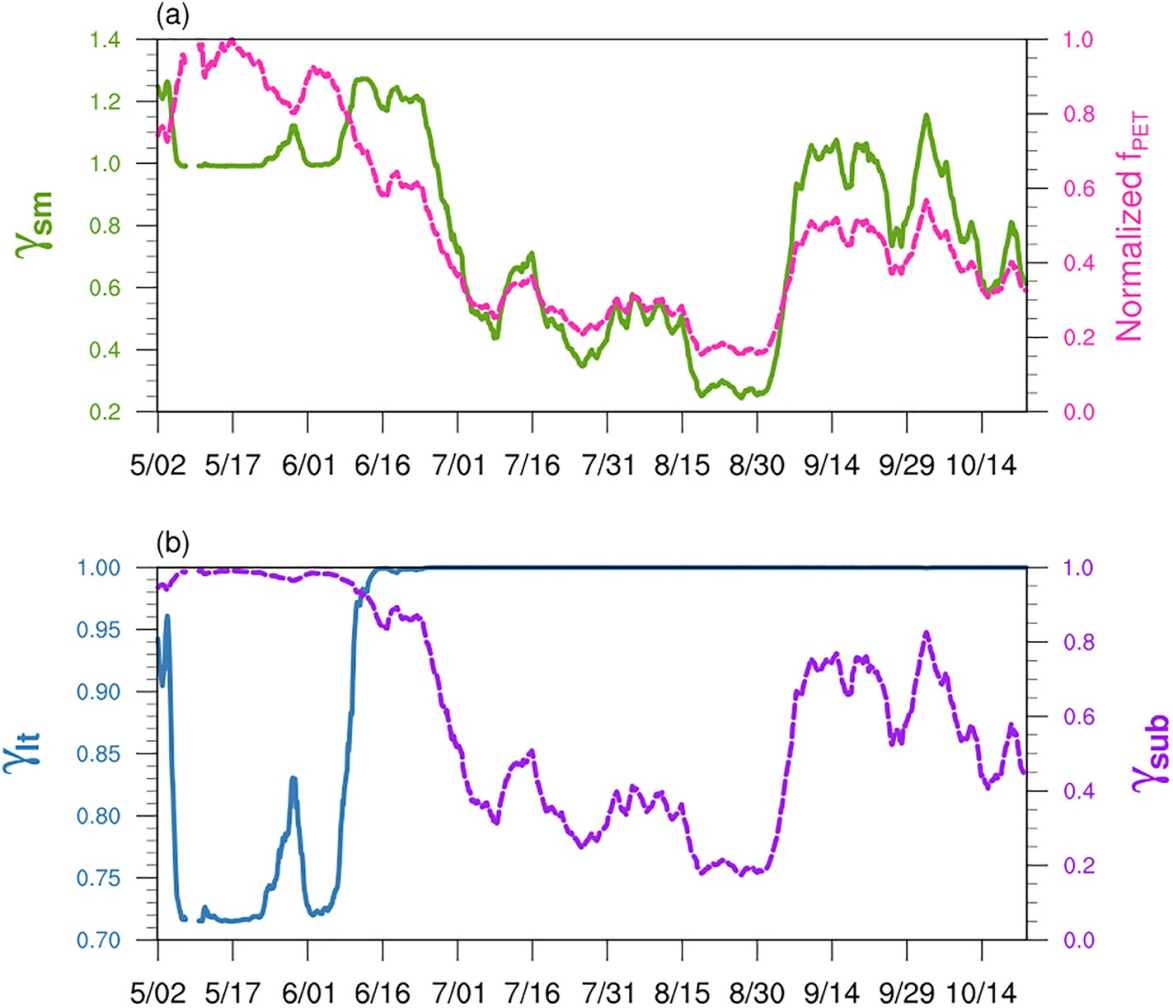
The impact of drought simulated by the offline PDS algorithm at the MOFLUX site in 2012. The total impact of drought (*γ*_sm_) on isoprene emission (green solid line) and the normalized 7‐day running averaged *f*_PET_ (pink dashed line) are presented in (a). The indirect impact of drought caused by stimulating temperature (*γ*_lt_, blue solid line) and the direct impact of drought caused by affecting substrate availability on isoprene emission (*γ*_sub_, purple dashed line) are presented in (b).

In the offline model, the two mechanisms are represented by *γ*_sub_ and *γ*_lt_. As shown in Figure 6, the onset of drought causes *γ*_lt_, which represents the indirect impact of drought, to increase and then stay stable. Meanwhile, the direct impact is controlled by the severity of drought with *γ*_sub_ decreasing with normalized drought index. Based on observations at the MOFLUX site, we assign a maximum of a 40% increase of isoprene emission (*γ*_sm_max_ = 1.4) due to the increase in leaf temperature. After combining with the direct impact of drought (*γ*_sub_), the maximum value of *γ*_sm_ is about 1.27, which results in an up to 27% increase in isoprene emission due to drought. Therefore, with the development of drought, the simulated *γ*_sm_ will initially increase and then decrease due to severe drought (Figure 6).

### 4.3 Global Scale Simulations

The drought algorithms were scaled up with global simulations using the FCSD component set (https://www.cesm.ucar.edu/models/cesm2/config/compsets.html) in the Community Earth System Model Version 2.1.3 (CESM2) (Danabasoglu et al., 2020). This was accomplished by integrating the drought response into the MEGAN component of the CLM‐CAM‐chem model and using this to simulate the impact of drought on isoprene emission and atmospheric chemistry in 2012 as an example. The model system was driven by the Modern‐Era Retrospective analysis for Research and Applications Version 2 (MERRA 2) reanalysis data set with about 1‐degree spatial resolution. The BVOC emissions were calculated by MEGAN embedded in the CLM 5 model with the prescribed satellite vegetation phenology. Anthropogenic and biomass burning emissions are obtained from the standard Coupled Model Intercomparison Project round 6 (CMIP6) (Eyring et al., 2016). Three model experiments were performed to test the drought algorithms: (a) without any drought algorithm, (b) with the online EDS drought algorithm, and (c) with the offline PDS drought algorithm. When we applied the offline PDS algorithm to the CLM, we modified Equation 7 and removed the impact of drought on stomatal conductance by deleting *β*_*t*_ from Equation 7, which means that the stomatal conductance could not be changed by the water stress function *β*_*t*_. The input for the offline model is the water stress function *β*_*t*_, which is also a normalized drought indicator in the range of 0–1 and is close to the normalized *f*_PET_ (Figure S1 in Supporting Information S1) at the MOFLUX site. Both the online and offline models show a decrease of isoprene emission (Figure S2 in Supporting Information S1), with the EDS and PDS algorithms decreasing the global isoprene emission by 11.0% and 10.4%, respectively.

**Figure 7:**
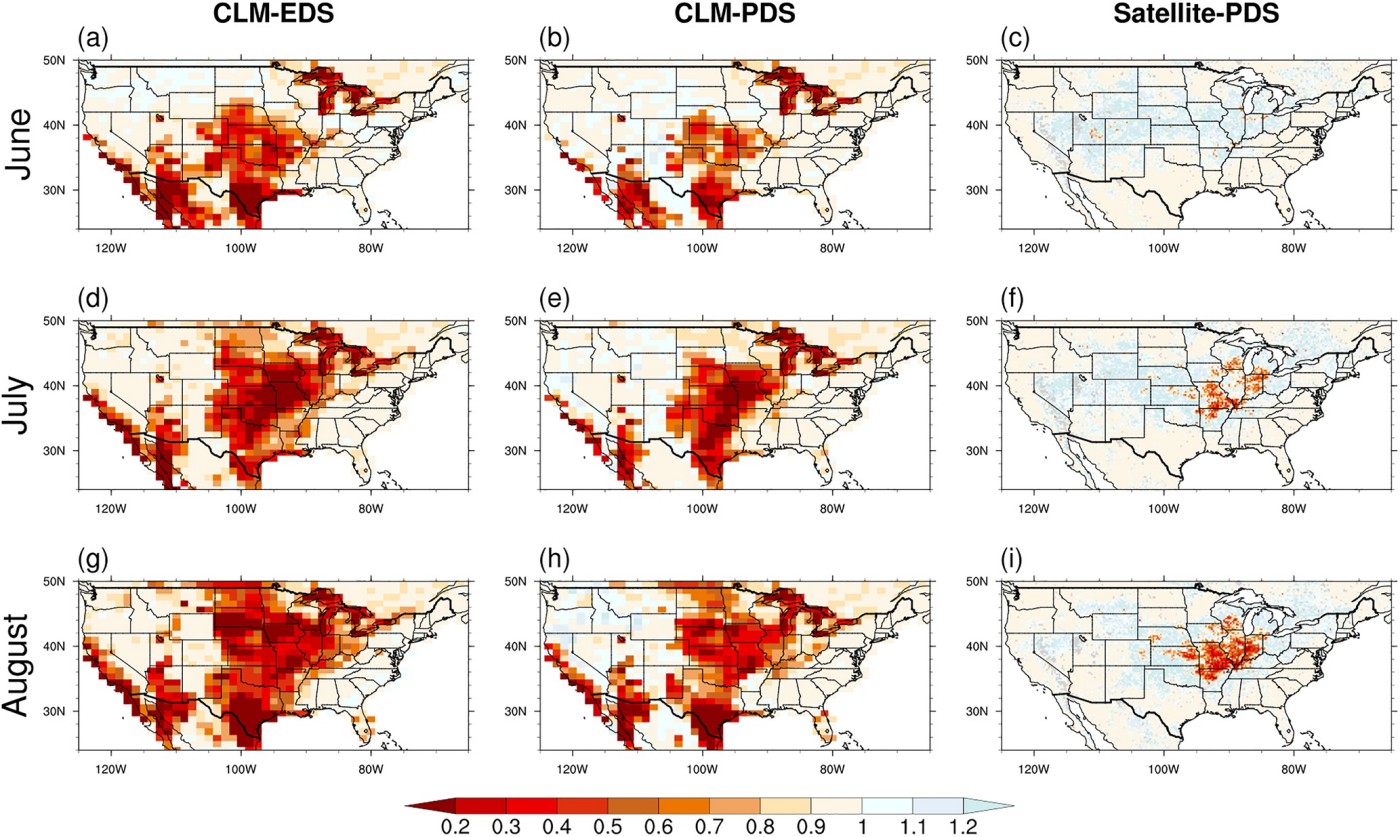
The spatial distributions of *γ*_sm_ calculated by the online Explicit Drought Stress (EDS) (first columns), the offline Parameterized Drought Stress (PDS) algorithms with *β*_*t*_ as inputs (second column) and with the satellite evaporative stress index (ESI) as inputs (third column). The three rows represent different months from June to August.

**Figure 8:**
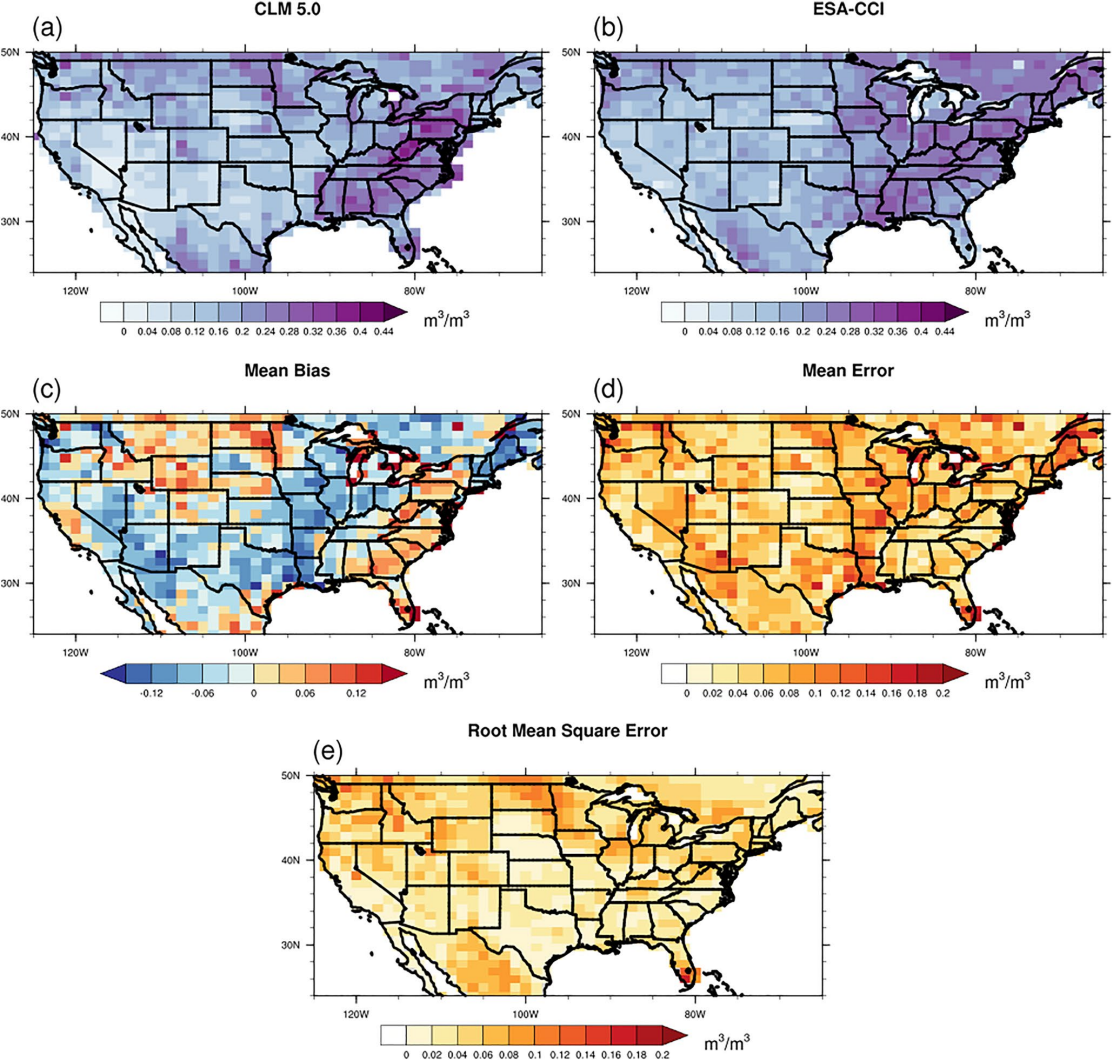
Comparison of the monthly surface soil moisture from the CLM model (a) and the ESA‐CCI data set (b) during July to August in 2012. The spatial distributions of the mean bias (c), the mean error (d) and the root mean square error (e) are also shown expressed in m^3^ m^−3^.

The offline PDS drought algorithm can also employ other drought indexes besides using *β*_*t*_ from the CLM model. This feature is useful for developing BVOC emission estimates for regional air quality simulations using readily available model inputs. This includes calculating the offline *γ*_sm_ using the ESI drought index. The 12‐week ESI index was normalized by the values of −3.5 and −0.5 to the range of 0–1. We compared the *γ*_sm_ calculated by the different combinations of algorithms and inputs for the CONtiguous United States (CONUS) region during the summer of 2012 as shown in Figure 7, and the inputs, *β*_*t*_ and the normalized ESI, are shown in Figure S3 in Supporting Information S1. The propagation of *γ*_sm_ from the satellite input is shown in Figure S4 in Supporting Information S1, and *γ*_sm_ from the satellite input reflects the impact of drought among the regions around Missouri, Illinois and Indiana states, but the spatial distribution of *γ*_sm_ derived from the CLM model shows a horizontally wider impact of drought. The *γ*_sm_ derived from the CLM model also shows the impact of drought in arid or semi‐arid regions like Texas and Arizona. One potential reason is that the CLM model overestimates the severity of drought. As shown in Figure 8, we compared the surface moisture simulated by CLM 5 with the ESA‐CCI satellite SM data sets, and the CLM model shows an underestimation of the surface SM with a negative MB during the summer of 2012 in the regions where the drought occurred. This indicates that the impact of drought might be exaggerated by the CLM model. Therefore, the skill of the land model to capture the drought behavior directly affects the simulated drought influence on isoprene emission.

**Figure 9:**
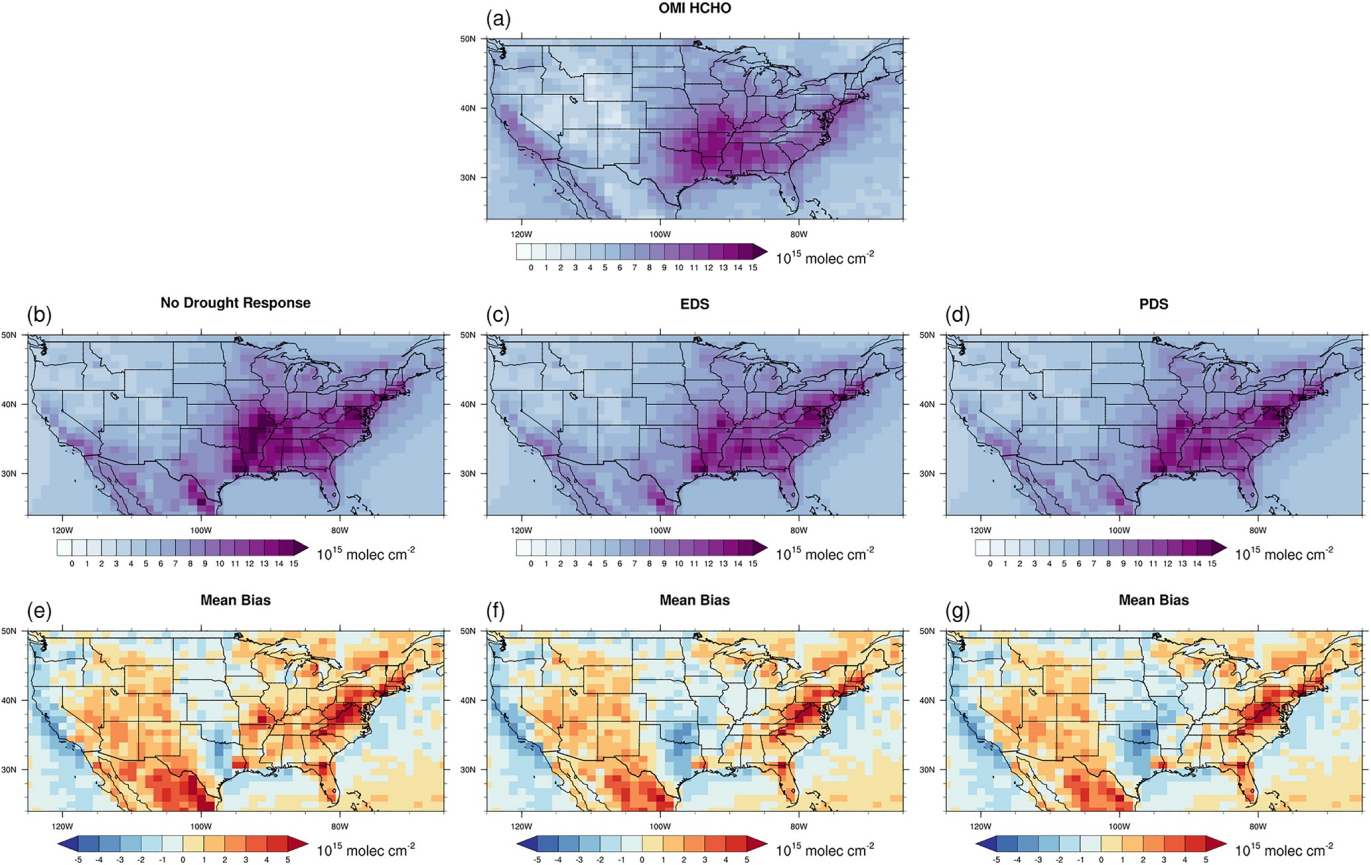
Comparison of the monthly OMI formaldehyde vertical column densities and the simulated formaldehyde vertical column densities by CAM‐chem in the CONUS region during May‐September 2012. The results by the no drought response experiment, the online Explicit Drought Stress (EDS) and the offline Parameterized Drought Stress (PDS) algorithm experiments are presented in the first, second and third columns, respectively. (a–d) show the spatial distribution of the averaged formaldehyde vertical column densities by OMI and CAM‐chem model with different drought treatment. The spatial distributions of the Mean Bias are also presented with the unit of 10^15^ molecules cm^−2^.

**Figure 10:**
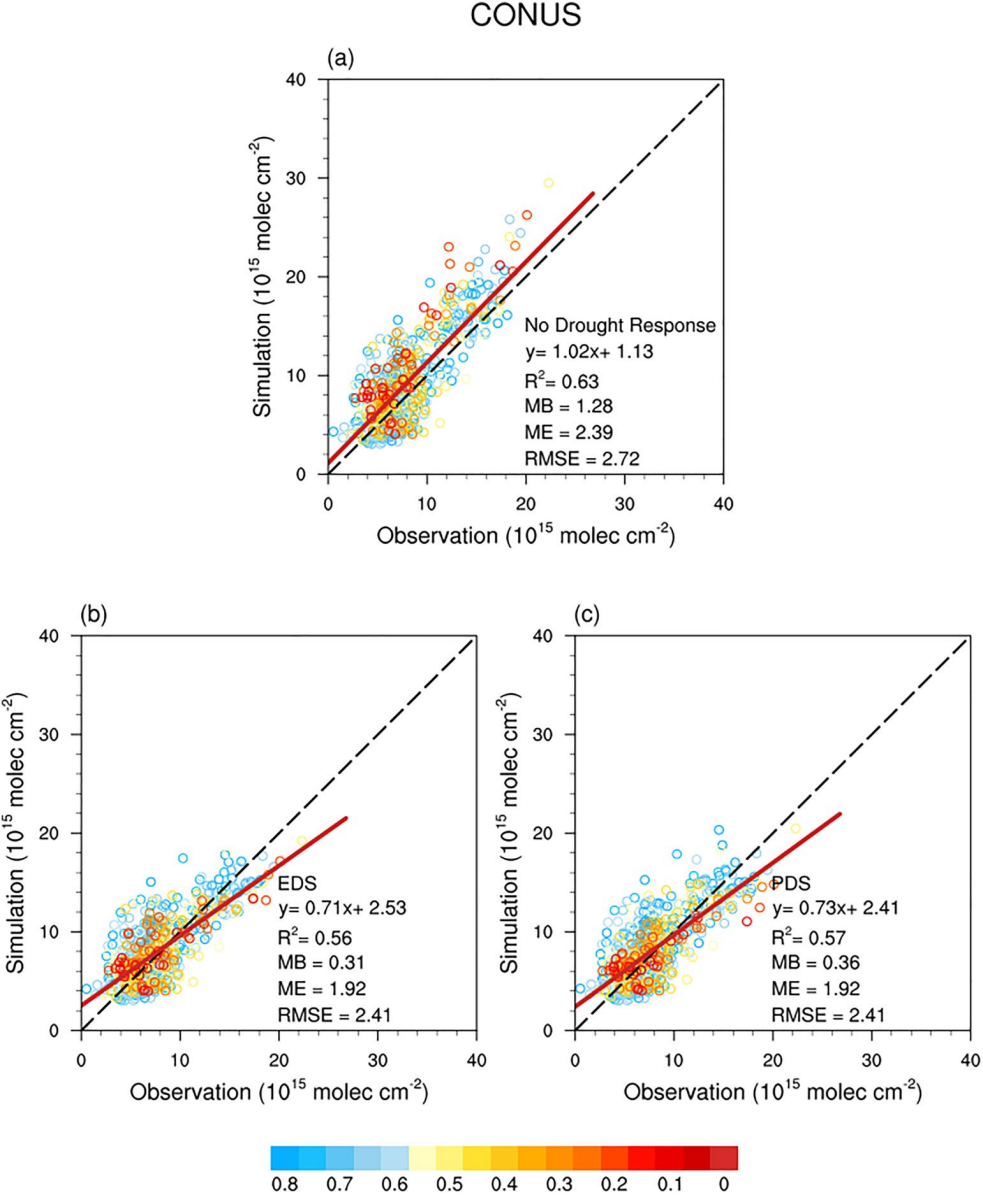
Comparisons between the monthly OMI formaldehyde vertical column densities and the simulated formaldehyde vertical column densities by CAM‐chem in the CONtiguous United States (CONUS). The results by the online Explicit Drought Stress (EDS) and the offline Parameterized Drought Stress (PDS) algorithms are presented in (b) and (c), respectively. The color of the points represents the value of the monthly *β*_*t*_. The mean bias (MB), the mean error (ME) and the root mean square error (RMSE) are presented with the unit of 10^15^ molecules cm^−2^.

We compared the monthly OMI HCHO vertical column densities with the modeled HCHO vertical column densities. The horizontal distribution of the HCHO vertical column densities for CONUS from the models and the satellite are compared in Figure 9 and show that the drought algorithms decrease the MB of simulated HCHO vertical column densities in the regions including the Missouri, Illinois, Arkansas, and Indiana states where the drought occurred in 2012 (Figure 9). However, the drought algorithms also increased the simulation errors in Oklahoma and Texas because the land model exaggerated the severity of drought in these two regions as shown in Figures 7 and 8. We also assessed the grids where tree cover fraction is greater than 30% and *β*_*t*_ is less than 0.85 in CONUS during May to September in 2012. As shown in Figure 10, the drought algorithms had a negative impact on the R^2^ and decreased the slope. The models assuming no drought affects shows an overestimation of HCHO vertical column density in CONUS in the high concentration regime, and the implementation of drought algorithms results in a better agreement with the observations as shown in Figure 10. The MB, ME, and RMSE in CONUS decreased from 1.28, 2.39, and 2.72 × 10^15^ molecules cm^−2^ to 0.31, 1.92, and 2.41 × 10^15^ molecules cm^−2^, respectively, with the online EDS drought algorithm and to 0.36, 1.92, and 2.41 × 10^15^ molecules cm^−2^, respectively, with the offline PDS drought algorithm. The results show that the drought algorithms could decrease the model biases in simulating the HCHO in drought regions. However, our assessment could also be affected by uncertainties from other sources besides the drought algorithms and the biogenic emission model. Therefore, more in‐situ observations, especially long‐term isoprene flux measurements, are required to fully validate the isoprene drought response algorithms for future predictions.

### 4.4 Comparison With Previous Studies

**Figure 11:**
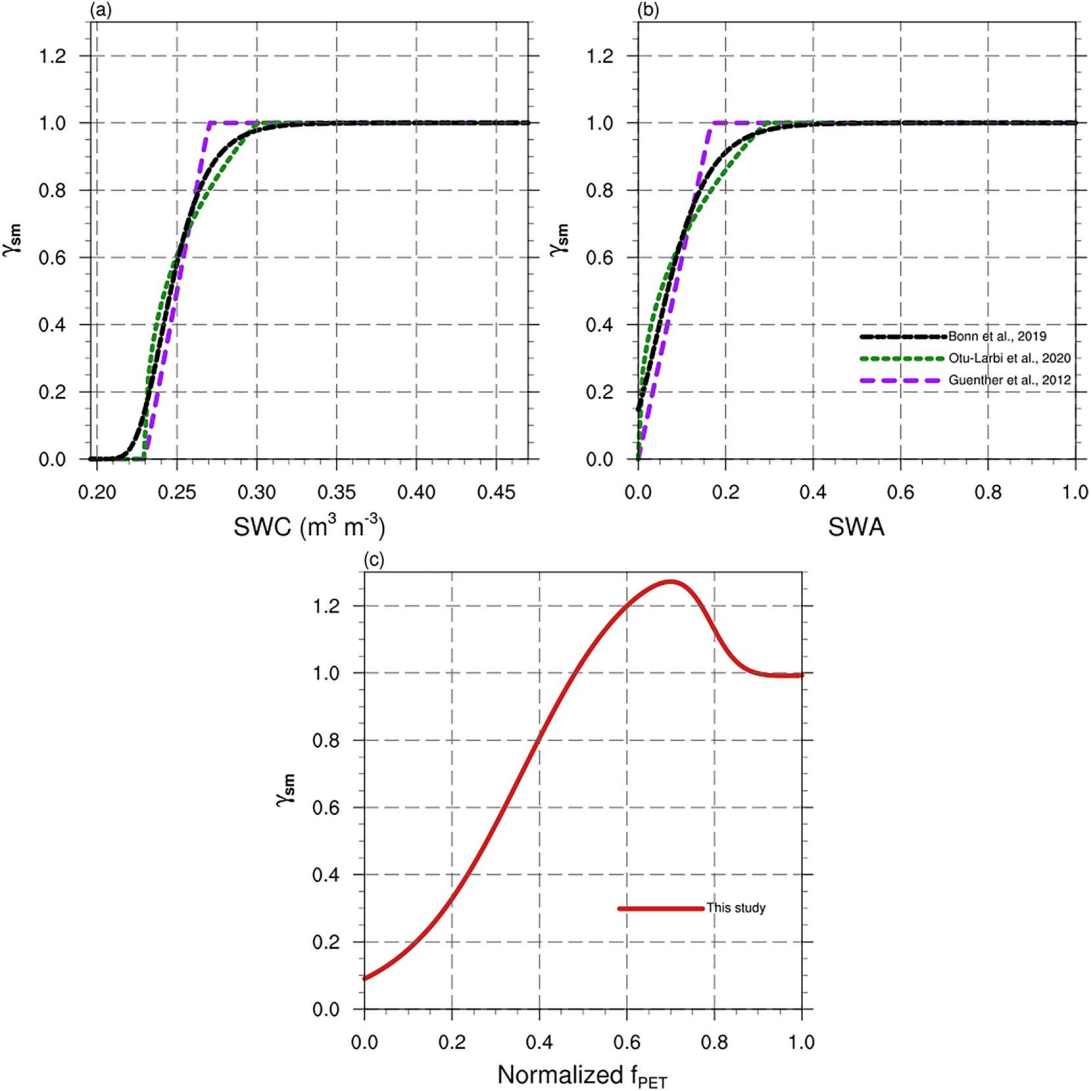
Comparison of drought algorithms with soil water content (SWC) as inputs (a) and with the normalized soil water availability (SWA) as inputs (b). (c) Presents the model proposed in this study with the normalized *f*_PET_ as inputs.

As shown in Table 2, previous drought algorithms for isoprene emission have mostly been based on volumetric soil water content (*θ* or SWC) or other SM‐relevant parameters like SWA because of the relatively easy access to the data (Bonn et al., 2019). Two threshold values of soil water content, the wilting point (*θ*_*w*_) and the critical SM (θ_c_), play a key role in these algorithms to define the severity of drought and the calculation of *γ*_sm_ (Bonn et al., 2019; Guenther et al., 2012; Otu‐Larbi et al., 2020). The assumption among these algorithms is that isoprene emission would not be affected when *θ* ≥ *θ*_*c*_. When the SM is in the range of *θ*_*w*_ < *θ* < *θ*_*c*_, *γ*_sm_ would decrease with the SM and reach 0 when the SM reaches the wilting point. The value of *γ*_sm_ is always in the range of 0–1, with lower values accounting for the negative impact of drought on isoprene emission by cutting off the supply of the carbon substrate for isoprene synthase. Bonn et al. (2019) uses the SWA index to replace SWC as input for the algorithm, but the SWA is also based on SM and the wilting point. SWA can be normalized to SWC with the range of 0–1 and is calculated as:

(14) $$\begin{array}{c} SWA=\frac{\theta -\theta_{w}}{\theta_{\max}-\theta_{w}} \end{array}$$

where *θ*_max_ and *θ*_*w*_ denote the maximum value of SWC and the wilting point, respectively. We compared the SM‐based algorithms as shown in Figure 11. The wilting point at the MOFLUX site is about 0.23 m^3^ m^−3^ according to Seco et al. (2015), and the maximum measured SWC was 0.47 m^3^ m^−3^ at the MOFLUX site during 2012. As shown in Figure 11 and Table 2, the previous SM algorithms follow the assumptions that we mentioned above, and the differences are the shape of the curve and the values of Δθ_1_, which is the difference between θ_w_ and θ_c_. Therefore, these algorithms are sensitive to the threshold values, as noted in previous studies (Bonn et al., 2019; Huang et al., 2015; Müller et al., 2008; Potosnak et al., 2014; Seco et al., 2015). We also presented these algorithms with the normalized inputs of SWA in Figure 11. These algorithms simulate lower isoprene when the SWA is below 0.4 because the isoprene emission is affected when the SM is near or below the wilting point. In addition, the thresholds of the algorithms differ because of different Δ*θ*_1_. In this study, we adopted the normalized *f*_PET_ as the model input for the offline algorithms. Because *f*_PET_ is a relatively more direct reflection of the water stress on the ecosystem, the impact of drought on isoprene emission during different stages may be better represented by *f*_PET_. That is, at the early stage of drought, the isoprene emission will not be affected by drought, and from the moderate stage to the severe stage of drought (*f*_PET_ < 0.9), the drought will initially increase isoprene emission due to an increase in leaf temperature and then decrease the isoprene emission due to the limited supply of the substrate as photosynthesis rates decline.

**Table 2:** Drought Algorithms Used for Simulating Isoprene Emission in the Previous and This Studies

Equation	Parameters	Inputs	Reference
$\begin{array}{c} \left\{\begin{array}{c} \gamma_{SM}=1\left(\theta \geq \theta_{c}\right)\\ \gamma_{SM}=\left(\theta -\theta_{w}\right)/∆\theta_{1}\\ \gamma_{SM}=0\left(\theta \leq \theta_{w}\right) \end{array}\right.\;\left(\theta_{w}<\theta <\theta_{c}\right)\# \end{array}$	*θ*_*w*_: Wilting point	*θ*: Volumetric soil water content	Guenther et al. (2012)
	*θ*_1_: Threshold (=*θ*_*w*_ + Δ*θ*_1_)		
	Δ*θ*_1_: 0.04 m^3^ m^−3^ (Guenther et al., 2012); 0.06 m^3^ m^−3^ (Guenther et al., 2006)		
$\begin{array}{c} \left\{\begin{array}{c} \gamma_{SM}=1\;\left(\beta_{t}\geq 0.6\right)\\ \gamma_{SM}=V_{c\;\max}/\alpha \;\left(0<\beta_{t}<0.6\right)\\ \gamma_{SM}=0\left(\beta_{t}=0\right) \end{array}\right. \end{array}$	*α*:37	*β*_*τ*_: Water stress function	Jiang et al. (2018)
		Vcmax: the maximum rate of carboxylation by the photosynthesis enzyme Rubisco	
$\begin{array}{c} \left\{\begin{array}{c} \gamma_{SM}=1\left(\theta \geq \theta_{c}\right)\\ \gamma_{SM}={\left[\left(\theta -\theta_{w}\right)/\left(\theta_{c}-\theta_{w}\right)\right]}^{0.4}\\ \gamma_{SM}=0\left(\theta \leq \theta_{w}\right) \end{array}\right.\;\left(\theta_{w}<\theta <\theta_{c}\right)\# \end{array}$	*θ*_*w*_: Wilting point	*θ*: Volumetric soil water content	Otu‐Larbi et al. (2020)
	*θ*_*c*_: Threshold (=*θ*_*w*_ + Δ*θ*_1_)		
	Δ*θ*_1_: 0.07 m^3^ m^−3^		
$\gamma_{SM}=\exp\left(-\exp\left(\left(0.056\right)\times \exp\left(1\right)\times \left(-2.3-SWA\right)+1\right)\right)$	–	SWA: Soil water availability	Bonn et al. (2019)
$\gamma_{SM}=\gamma_{sm\_max}\left(\frac{1}{1+b_{1}\cdot e^{a_{1}\cdot \left(K_{c}-0.2\right)}}\right)\left(\frac{1}{\gamma_{sm\_max}}+\frac{\left(1-\frac{1}{\gamma_{sm\_\max}}\right)}{1+b_{2}\cdot e^{a_{2}\cdot \left(1.3-K_{c}\right)}}\right)$	*γ*_sm_max_: 1.4	*f*_PET_: Normalized drought indicator	This study

**Figure 12:**
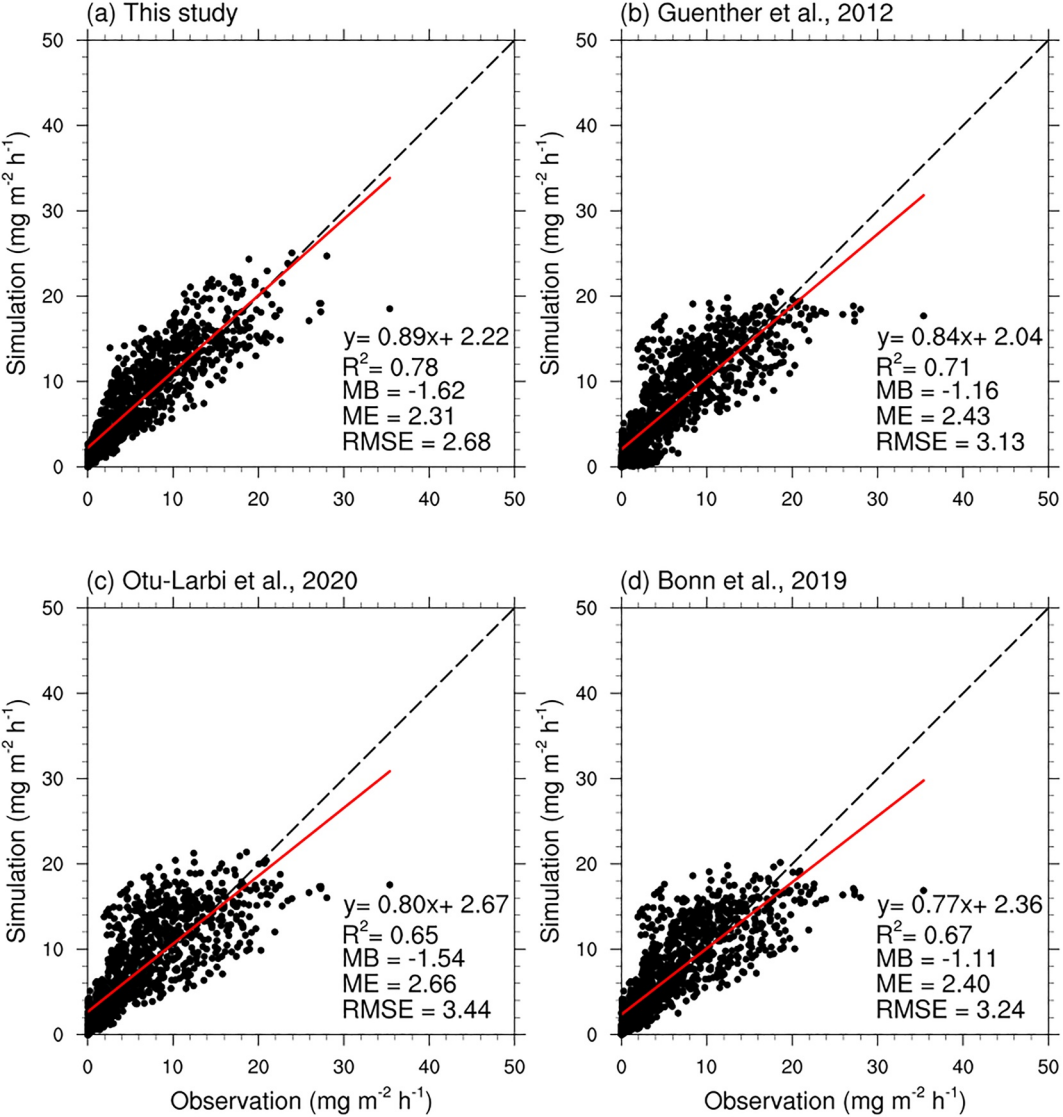
Scatter plots of measured diurnal isoprene fluxes and modeled daily isoprene fluxes with different drought algorithms and a wilting point of 0.196 m^3^ m^−3^.

We applied each of the SM‐based algorithms listed in Table 2 for the MOFLUX site during 2012. The default wilting point data set used for MEGANv2 and v2.1 comes from the global database of Chen and Dudhia (2001) and has a wilting point of 0.084 m^3^ m^−3^ for the MOFLUX site. The models listed in Table 2 will never have any drought impacts with a wilting point of 0.084 m^3^ m^−3^ because the θ_w_ and θ_c_ are always below the observed SM levels. Therefore, we used the value of 0.23 m^3^ m^−3^ recommended by Seco et al. (2015) and based on site characteristics. However, the previous SM‐based algorithms did not capture the variability of isoprene flux (Figure S5 in Supporting Information S1). We also tested the case of using the minimum value of SM (0.196 m^3^ m^−3^) as the wilting point at the MOFLUX site (Figure 12), and the results showed better agreement with the observations than the results with the wilting point of 0.23 m^3^ m^−3^ (Figure S5 in Supporting Information S1). The choice of θ_w_, or the thresholds, overwhelmingly determines the performance of these algorithms. The SM input data, which is difficult to simulate in global models, will also affect the performance of these algorithms. In our experiments, we used the in situ SM measurements at 10 cm depth as the model input. There is a strong vertical gradient in SM so the soil depth where measurements are made, or modeled values predicted, could also affect model performance. If the input is changed to other SM data sets for different depths, such as the surface SM data sets from satellite or root zone SM data sets, the current threshold values would not be appropriate (Opacka et al., 2022).

### 4.5 Future Direction

Heatwaves and drought often happen simultaneously and both can influence isoprene emission. Ferracci et al. (2020) and Potosnak et al. (2014) both observed a higher than expected increase of isoprene concentration and flux during mild drought and heatwave events. Recent evidence from the satellite HCHO observation also shows an increase of isoprene during the drought and heatwave events (Morfopoulos et al., 2022). It is difficult to distinguish any individual impacts of these two processes directly from the in‐situ observations. Besides the *γ*_sm_, Otu‐Larbi et al. (2020) introduced another independent correction factor to explain the impact of high temperature. In the framework of MEGAN, the impact of heatwaves on isoprene emission is currently not considered independently, and the impact of high temperature is described by the temperature response algorithm. In addition to the impact of the current temperature, MEGAN also considers the influence of past temperature. This includes the average temperature of the past 24 hours and the average temperature of the past 10 days. As shown in Figure 4 of Guenther et al. (2006), elevated temperature for the preceding days would also increase isoprene emission in the temperature response algorithm. Otu‐Larbi et al. (2020) used the original temperature algorithm of Guenther et al. (1993) that only accounts for the current temperature that represents the instantaneous isoprene synthase enzyme activity. Besides the impact of the past high temperature during a heat wave, another factor that could increase the isoprene emission is the drought impact on the leaf temperature, which has been considered in this study and was estimated to reach an increase of 27% in the offline PDS drought algorithm and reach a maximum increase of 14.5% caused by a ∼0.84°C temperature change in the online EDS algorithm. However, this is based on the leaf temperature results simulated by the CLM model, which is subject to model uncertainties, including the stomatal conductance algorithm. Field and laboratory experiments have shown that water stress could increase the leaf temperature to a much higher level of about 3°–4°C under high photosynthetically active radiation (Gerhards et al., 2016; Reynolds‐Henne et al., 2010). Therefore, there is a need for further investigation of the connections among drought, stomatal conductance, leaf temperature, ET, and isoprene emission using observations and modeling.

In addition, isoprene emission drought response studies are still limited by having canopy scale flux observations only from a single site and there is an urgent need for more observations. For example, at the same MOFLUX site, Geron et al. (2016) found that the tree species with diverse tolerance of water stress show different reactions of isoprene emission to drought. The purpose of this study is to establish new model frameworks for simulating the impact of drought, and the further validation and improvements require more observations. This include more observations at deciduous broadleaf forests as well as observations of the responses of other ecosystem, and it could be important to include isohydric as well as anisohydric plant species. The flux measurements are currently rare due to the expense and the measurements that focus on drought and heatwave are difficult to obtain. Relaxed eddy accumulation technique (Ciccioli et al., 2003; Sarkar et al., 2020) combined with a gas chromatograph with photoionization detection (Bolas et al., 2020) is an example of a lower‐cost alternative for isoprene flux measurements and create more data for model validation and improvement. Besides having more in‐situ ground observations, the existing and future airborne observations and satellite products could also provide an opportunity to further investigate and understand the impact of environmental stress on BVOC emission. The high resolution (30–70 m) ET and ESI index products from the ECOsystem Spaceborne Thermal Radiometer Experiment on Space Station (ECOSTRESS) could be a good tool for monitoring water stress and providing model inputs. In addition, the high resolution HCHO observations from the TROPOspheric Monitoring Instrument (TROPOMI) instrument (Veefkind et al., 2012) and recent direct observations of isoprene from the Cross‐track Infrared Sounder (CrIS) instrument (Fu et al., 2019; Wells et al., 2020) may also provide information on atmospheric chemistry processes and improve assessments of the impact of drought.

## Supporting information

Supporting Information S1

## Data Availability

The Community Land Model and Community Atmospheric Model with Chemistry are available on the website of the Community Earth System Model (CESM) v2 (https://www.cesm.ucar.edu/models/cesm2/). The independent MEGAN v3.2 are available through the link: https://bai.ess.uci.edu/megan.

## References

[jame21739-bib-0001] Anderson, M. C. , Hain, C. , Otkin, J. , Zhan, X. , Mo, K. , Svoboda, M. , et al. (2013). An intercomparison of drought indicators based on thermal remote sensing and NLDAS‐2 simulations with US Drought Monitor classifications. Journal of Hydrometeorology, 14(4), 1035–1056. 10.1175/jhm-d-12-0140.1

[jame21739-bib-0002] Anderson, M. C. , Hain, C. , Wardlow, B. , Pimstein, A. , Mecikalski, J. R. , & Kustas, W. P. (2011). Evaluation of drought indices based on thermal remote sensing of evapotranspiration over the continental United States. Journal of Climate, 24(8), 2025–2044. 10.1175/2010jcli3812.1

[jame21739-bib-0003] Arneth, A. , Niinemets, Ü. , Pressley, S. , Bäck, J. , Hari, P. , Karl, T. , et al. (2007). Process‐based estimates of terrestrial ecosystem isoprene emissions: Incorporating the effects of a direct CO 2‐isoprene interaction. Atmospheric Chemistry and Physics, 7(1), 31–53. 10.5194/acp-7-31-2007

[jame21739-bib-0004] Bolas, C. G. , Ferracci, V. , Robinson, A. D. , Mead, M. I. , Nadzir, M. S. M. , Pyle, J. A. , et al. (2020). iDirac: A field‐portable instrument for long‐term autonomous measurements of isoprene and selected VOCs. Atmospheric Measurement Techniques, 13(2), 821–838. 10.5194/amt-13-821-2020

[jame21739-bib-0005] Bonn, B. , Magh, R. K. , Rombach, J. , & Kreuzwieser, J. (2019). Biogenic isoprenoid emissions under drought stress: Different responses for isoprene and terpenes. Biogeosciences, 16(23), 4627–4645. 10.5194/bg-16-4627-2019

[jame21739-bib-0006] Brilli, F. , Barta, C. , Fortunati, A. , Lerdau, M. , Loreto, F. , & Centritto, M. (2007). Response of isoprene emission and carbon metabolism to drought in white poplar (Populus alba) saplings. New Phytologist, 175(2), 244–254. 10.1111/j.1469-8137.2007.02094.x 17587373

[jame21739-bib-0007] Brüggemann, N. , & Schnitzler, J. P. (2002). Comparison of isoprene emission, intercellular isoprene concentration and photosynthetic performance in water‐limited oak (quercus pubescens willd. And quercus robur L.) saplings. Plant Biology, 4, 456–463. 10.1055/s-2002-34128

[jame21739-bib-0008] Chen, F. , & Dudhia, J. (2001). Coupling an advanced land surface–hydrology model with the penn state–NCAR MM5 modeling system. Part I: Model implementation and sensitivity. Monthly Weather Review, 129(4), 569–585. 10.1175/1520-0493(2001)129<0569:CAALSH>2.0.CO;2

[jame21739-bib-0009] Chen, W. H. , Guenther, A. B. , Wang, X. M. , Chen, Y. H. , Gu, D. S. , Chang, M. , et al. (2018). Regional to global biogenic isoprene emission responses to changes in vegetation from 2000 to 2015. Journal of Geophysical Research: Atmospheres, 123(7), 3757–3771. 10.1002/2017JD027934

[jame21739-bib-0010] Ciccioli, P. , Brancaleoni, E. , Frattoni, M. , Marta, S. , Brachetti, A. , Vitullo, M. , et al. (2003). Relaxed eddy accumulation, a new technique for measuring emission and deposition fluxes of volatile organic compounds by capillary gas chromatography and mass spectrometry. Journal of Chromatography A, 985(1), 283–296. 10.1016/S0021-9673(02)01731-4 12580496

[jame21739-bib-0011] Claeys, M. , Graham, B. , Vas, G. , Wang, W. , Vermeylen, R. , Pashynska, V. , et al. (2004). Formation of secondary organic aerosols through photooxidation of isoprene. Science, 303(5661), 1173–1176. 10.1126/science.1092805 14976309

[jame21739-bib-0012] Collatz, G. J. , Ball, J. T. , Grivet, C. , & Berry, J. A. (1991). Physiological and environmental regulation of stomatal conductance, photosynthesis and transpiration: A model that includes a laminar boundary layer. Agricultural and Forest Meteorology, 54(2–4), 107–136. 10.1016/0168-1923(91)90002-8

[jame21739-bib-0013] Computational and Information Systems Laboratory . (2019). Cheyenne: HPE/SGI ICE XA system (university community computing). National Center for Atmospheric Research. 10.5065/D6RX99HX

[jame21739-bib-0014] Danabasoglu, G. , Lamarque, J. F. , Bacmeister, J. , Bailey, D. A. , DuVivier, A. K. , Edwards, J. , et al. (2020). The community Earth system model version 2 (CESM2). Journal of Advances in Modeling Earth Systems, 12, e2019MS001916. 10.1029/2019MS001916

[jame21739-bib-0015] De Smedt, I. , Stavrakou, T. , Hendrick, F. , Danckaert, T. , Vlemmix, T. , Pinardi, G. , et al. (2015). Diurnal, seasonal and long‐term variations of global formaldehyde columns inferred from combined OMI and GOME‐2 observations. Atmospheric Chemistry and Physics, 15(21), 12519–12545. 10.5194/acp-15-12519-2015

[jame21739-bib-0016] De Smedt, I. , Van Roozendael, M. , Stavrakou, T. , Müller, J. F. , Lerot, C. , Theys, N. , et al. (2012). Improved retrieval of global tropospheric formaldehyde columns from GOME‐2/MetOp‐A addressing noise reduction and instrumental degradation issues. Atmospheric Measurement Techniques, 5(11), 2933–2949. 10.5194/amt-5-2933-2012

[jame21739-bib-0017] Dorigo, W. , Wagner, W. , Albergel, C. , Albrecht, F. , Balsamo, G. , Brocca, L. , et al. (2017). ESA CCI Soil Moisture for improved Earth system understanding: State‐of‐the art and future directions. Remote Sensing of Environment, 203, 185–215. 10.1016/j.rse.2017.07.001

[jame21739-bib-0018] Duncan, B. N. , Yoshida, Y. , Damon, M. R. , Douglass, A. R. , & Witte, J. C. (2009). Temperature dependence of factors controlling isoprene emissions. Geophysical Research Letters, 36(5), L05813. 10.1029/2008GL037090

[jame21739-bib-0019] Emmerson, K. M. , Palmer, P. I. , Thatcher, M. , Haverd, V. , & Guenther, A. B. (2019). Sensitivity of isoprene emissions to drought over south‐eastern Australia: Integrating models and satellite observations of soil moisture. Atmospheric Environment, 209, 112–124. 10.1016/j.atmosenv.2019.04.038

[jame21739-bib-0020] Emmons, L. K. , Schwantes, R. H. , Orlando, J. J. , Tyndall, G. , Kinnison, D. , Lamarque, J.‐F. , et al. (2020). The chemistry mechanism in the community Earth system model version 2 (CESM2). Journal of Advances in Modeling Earth Systems, 12(4), e2019MS001882. 10.1029/2019MS001882

[jame21739-bib-0021] Eyring, V. , Bony, S. , Meehl, G. A. , Senior, C. A. , Stevens, B. , Stouffer, R. J. , & Taylor, K. E. (2016). Overview of the coupled model intercomparison project phase 6 (CMIP6) experimental design and organization. Geoscientific Model Development, 9(5), 1937–1958. 10.5194/gmd-9-1937-2016

[jame21739-bib-0022] Fang, C. , Monson, R. K. , & Cowling, E. B. (1996). Isoprene emission, photosynthesis, and growth in sweetgum (Liquidambar styraciflua) seedlings exposed to short‐ and long‐term drying cycles. Tree Physiology, 16(4), 441–446. 10.1093/treephys/16.4.441 14871730

[jame21739-bib-0023] Ferracci, V. , Bolas, C. G. , Freshwater, R. A. , Staniaszek, Z. , King, T. , Jaars, K. , et al. (2020). Continuous isoprene measurements in a UK temperate forest for a whole growing season: Effects of drought stress during the 2018 heatwave. Geophysical Research Letters, 47(15), e2020GL088885. 10.1029/2020GL088885

[jame21739-bib-0024] Fortunati, A. , Barta, C. , Brilli, F. , Centritto, M. , Zimmer, I. , Schnitzler, J.‐P. , & Loreto, F. (2008). Isoprene emission is not temperature‐dependent during and after severe drought‐stress: A physiological and biochemical analysis. The Plant Journal, 55(4), 687–697. 10.1111/j.1365-313X.2008.03538.x 18445130

[jame21739-bib-0025] Fu, D. , Millet, D. B. , Wells, K. C. , Payne, V. H. , Yu, S. , Guenther, A. , & Eldering, A. (2019). Direct retrieval of isoprene from satellite‐based infrared measurements. Nature Communications, 10(1), 3811. 10.1038/s41467-019-11835-0 PMC6707292 31444348

[jame21739-bib-0026] Gerhards, M. , Rock, G. , Schlerf, M. , & Udelhoven, T. (2016). Water stress detection in potato plants using leaf temperature, emissivity, and reflectance. International Journal of Applied Earth Observation and Geoinformation, 53, 27–39. 10.1016/j.jag.2016.08.004

[jame21739-bib-0027] Geron, C. , Daly, R. , Harley, P. , Rasmussen, R. , Seco, R. , Guenther, A. , et al. (2016). Large drought‐induced variations in oak leaf volatile organic compound emissions during PINOT NOIR 2012. Chemosphere, 146, 8–21. 10.1016/j.chemosphere.2015.11.086 26706927

[jame21739-bib-0028] Gruber, A. , Scanlon, T. , van der Schalie, R. , Wagner, W. , & Dorigo, W. (2019). Evolution of the ESA CCI Soil Moisture climate data records and their underlying merging methodology. Earth System Science Data, 11(2), 717–739. 10.5194/essd-11-717-2019

[jame21739-bib-0029] Gu, L. , Pallardy, S. G. , Yang, B. , Hosman, K. P. , Mao, J. , Ricciuto, D. , et al. (2016). Testing a land model in ecosystem functional space via a comparison of observed and modeled ecosystem flux responses to precipitation regimes and associated stresses in a Central U.S. forest. Journal of Geophysical Research: Biogeosciences, 121(7), 1884–1902. 10.1002/2015JG003302

[jame21739-bib-0030] Guenther, A. , Karl, T. , Harley, P. , Wiedinmyer, C. , Palmer, P. , & Geron, C. (2006). Estimates of global terrestrial isoprene emissions using MEGAN (model of emissions of Gases and aerosols from nature). Atmospheric Chemistry and Physics, 6(11), 3181–3210. 10.5194/acp-6-3181-2006

[jame21739-bib-0031] Guenther, A. B. , Jiang, X. , Heald, C. L. , Sakulyanontvittaya, T. , Duhl, T. , Emmons, L. K. , & Wang, X. (2012). The model of emissions of Gases and aerosols from nature version 2.1 (MEGAN2.1): An extended and updated framework for modeling biogenic emissions. Geoscientific Model Development, 5(6), 1471–1492. 10.5194/gmd-5-1471-2012

[jame21739-bib-0032] Guenther, A. B. , Zimmerman, P. R. , Harley, P. C. , Monson, R. K. , & Fall, R. (1993). Isoprene and monoterpene emission rate variability: Model evaluations and sensitivity analyses. Journal of Geophysical Research, 98(D7), 12609–12617. 10.1029/93JD00527

[jame21739-bib-0033] Hanson, R. L. (1991). Evapotranspiration and droughts, national water summary 1988–89: Hydrologic events and floods and droughts US geological survey water‐supply (pp. 99–104). Paper 2375.

[jame21739-bib-0034] Huang, L. , McGaughey, G. , McDonald‐Buller, E. , Kimura, Y. , & Allen, D. T. (2015). Quantifying regional, seasonal and interannual contributions of environmental factors on isoprene and monoterpene emissions estimates over eastern Texas. Atmospheric Environment, 106, 120–128. 10.1016/j.atmosenv.2015.01.072

[jame21739-bib-0035] Jiang, X. , Guenther, A. , Potosnak, M. , Geron, C. , Seco, R. , Karl, T. , et al. (2018). Isoprene emission response to drought and the impact on global atmospheric chemistry. Atmospheric Environment, 183, 69–83. 10.1016/j.atmosenv.2018.01.026 30505205 PMC6260947

[jame21739-bib-0036] Kaiser, J. , Jacob, D. J. , Zhu, L. , Travis, K. R. , Fisher, J. A. , González Abad, G. , et al. (2018). High‐resolution inversion of OMI formaldehyde columns to quantify isoprene emission on ecosystem‐relevant scales: Application to the southeast US. Atmospheric Chemistry and Physics, 18(8), 5483–5497. 10.5194/acp-18-5483-2018

[jame21739-bib-0037] Kaser, L. , Peron, A. , Graus, M. , Striednig, M. , Wohlfahrt, G. , Juráň, S. , & Karl, T. (2022). Interannual variability of terpenoid emissions in an alpine city. Atmospheric Chemistry and Physics, 22(8), 5603–5618. 10.5194/acp-22-5603-2022

[jame21739-bib-0038] Morfopoulos, C. , Müller, J.‐F. , Stavrakou, T. , Bauwens, M. , De Smedt, I. , Friedlingstein, P. , et al. (2022). Vegetation responses to climate extremes recorded by remotely sensed atmospheric formaldehyde. Global Change Biology, 28(5), 1809–1822. 10.1111/gcb.15880 34510653 PMC9290652

[jame21739-bib-0039] Müller, J. F. , Stavrakou, T. , Wallens, S. , De Smedt, I. , Van Roozendael, M. , Potosnak, M. J. , et al. (2008). Global isoprene emissions estimated using MEGAN, ECMWF analyses and a detailed canopy environment model. Atmospheric Chemistry and Physics, 8(5), 1329–1341. 10.5194/acp-8-1329-2008

[jame21739-bib-0040] Niinemets, U. (2010). Mild versus severe stress and BVOCs: Thresholds, priming and consequences. Trends in Plant Science, 15(3), 145–153. 10.1016/j.tplants.2009.11.008 20006534

[jame21739-bib-0041] Novick, K. A. , Ficklin, D. L. , Stoy, P. C. , Williams, C. A. , Bohrer, G. , Oishi, A. C. , et al. (2016). The increasing importance of atmospheric demand for ecosystem water and carbon fluxes. Nature Climate Change, 6(11), 1023–1027. 10.1038/nclimate3114

[jame21739-bib-0042] Oleson, K. , Lawrence, D. , Bonan, G. , Drewniak, B. , Huang, M. , Koven, C. , et al. (2013). Technical description of version 4.5 of the community land model (CLM), NCAR Tech. Note (p. 420). NCAR/TN‐503+ STR. 10.5065/D6RR1W7M

[jame21739-bib-0043] Opacka, B. , Müller, J. F. , Stavrakou, T. , Bauwens, M. , Sindelarova, K. , Markova, J. , & Guenther, A. B. (2021). Global and regional impacts of land cover changes on isoprene emissions derived from spaceborne data and the MEGAN model. Atmospheric Chemistry and Physics, 21(11), 8413–8436. 10.5194/acp-21-8413-2021

[jame21739-bib-0044] Opacka, B. , Müller, J.‐F. , Stavrakou, T. , Miralles, D. G. , Koppa, A. , Pagán, B. R. , et al. (2022). Impact of drought on isoprene fluxes assessed using field data, satellite‐based GLEAM soil moisture and HCHO observations from OMI. Remote Sensing, 14(9), 2021. 10.3390/rs14092021

[jame21739-bib-0045] Otu‐Larbi, F. , Bolas, C. G. , Ferracci, V. , Staniaszek, Z. , Jones, R. L. , Malhi, Y. , et al. (2020). Modelling the effect of the 2018 summer heatwave and drought on isoprene emissions in a UK woodland. Global Change Biology, 26(4), 2320–2335. 10.1111/gcb.14963 31837069

[jame21739-bib-0046] Palmer, P. I. , Jacob, D. J. , Fiore, A. M. , Martin, R. V. , Chance, K. , & Kurosu, T. P. (2003). Mapping isoprene emissions over North America using formaldehyde column observations from space. Journal of Geophysical Research, 108(D6), 2002JD002153. 10.1029/2002JD002153

[jame21739-bib-0047] Park Williams, A. , Allen, C. D. , Macalady, A. K. , Griffin, D. , Woodhouse, C. A. , Meko, D. M. , et al. (2013). Temperature as a potent driver of regional forest drought stress and tree mortality. Nature Climate Change, 3, 292–297. 10.1038/nclimate1693

[jame21739-bib-0048] Pegoraro, E. , Rey, A. , Greenberg, J. , Harley, P. , Grace, J. , Malhi, Y. , & Guenther, A. (2004). Effect of drought on isoprene emission rates from leaves of Quercus virginiana Mill. Atmospheric Environment, 38(36), 6149–6156. 10.1016/j.atmosenv.2004.07.028

[jame21739-bib-0049] Porporato, A. , Laio, F. , Ridolfi, L. , & Rodriguez‐Iturbe, I. (2001). Plants in water‐controlled ecosystems: Active role in hydrologic processes and response to water stress: III. Vegetation water stress. Advances in Water Resources, 24(7), 725–744. 10.1016/S0309-1708(01)00006-9

[jame21739-bib-0050] Potosnak, M. J. , LeStourgeon, L. , Pallardy, S. G. , Hosman, K. P. , Gu, L. , Karl, T. , et al. (2014). Observed and modeled ecosystem isoprene fluxes from an oak‐dominated temperate forest and the influence of drought stress. Atmospheric Environment, 84, 314–322. 10.1016/j.atmosenv.2013.11.055

[jame21739-bib-0051] Reynolds‐Henne, C. E. , Langenegger, A. , Mani, J. , Schenk, N. , Zumsteg, A. , & Feller, U. (2010). Interactions between temperature, drought and stomatal opening in legumes. Environmental and Experimental Botany, 68(1), 37–43. 10.1016/j.envexpbot.2009.11.002

[jame21739-bib-0052] Sarkar, C. , Turnipseed, A. , Shertz, S. , Karl, T. , Potosnak, M. , Bai, J. , et al. (2020). A portable, low‐cost relaxed eddy accumulation (REA) system for quantifying ecosystem‐level fluxes of volatile organics. Atmospheric Environment, 242, 117764. 10.1016/j.atmosenv.2020.117764

[jame21739-bib-0053] Schulze, E. D. (1986). Carbon dioxide and water vapor exchange in response to drought in the atmosphere and in the soil. Annual Review of Plant Physiology, 37(1), 247–274. 10.1146/annurev.pp.37.060186.001335

[jame21739-bib-0054] Schwantes, R. H. , Emmons, L. K. , Orlando, J. J. , Barth, M. C. , Tyndall, G. S. , Hall, S. R. , et al. (2020). Comprehensive isoprene and terpene gas‐phase chemistry improves simulated surface ozone in the southeastern US. Atmospheric Chemistry and Physics, 20(6), 3739–3776. 10.5194/acp-20-3739-2020

[jame21739-bib-0055] Seco, R. , Holst, T. , Matzen, M. S. , Westergaard‐Nielsen, A. , Li, T. , Simin, T. , et al. (2020). Volatile organic compound fluxes in a subarctic peatland and lake. Atmospheric Chemistry and Physics, 20(21), 13399–13416. 10.5194/acp-20-13399-2020

[jame21739-bib-0056] Seco, R. , Karl, T. , Guenther, A. , Hosman, K. P. , Pallardy, S. G. , Gu, L. , et al. (2015). Ecosystem‐scale volatile organic compound fluxes during an extreme drought in a broadleaf temperate forest of the Missouri Ozarks (central USA). Global Change Biology, 21(10), 3657–3674. 10.1111/gcb.12980 25980459

[jame21739-bib-0057] Seco, R. , Karl, T. , Turnipseed, A. , Greenberg, J. , Guenther, A. , Llusia, J. , et al. (2017). Springtime ecosystem‐scale monoterpene fluxes from Mediterranean pine forests across a precipitation gradient. Agricultural and Forest Meteorology, 237–238, 150–159. 10.1016/j.agrformet.2017.02.007

[jame21739-bib-0058] Sellers, P. J. , Randall, D. A. , Collatz, G. J. , Berry, J. A. , Field, C. B. , Dazlich, D. A. , et al. (1996). A revised land surface parameterization (SiB2) for atmospheric GCMS. Part I: Model formulation. Journal of Climate, 9(4), 676–705. 10.1175/1520-0442(1996)009<0676:ARLSPF>2.0.CO;2

[jame21739-bib-0059] Sharkey, T. D. , & Loreto, F. (1993). Water stress, temperature, and light effects on the capacity for isoprene emission and photosynthesis of kudzu leaves. Oecologia, 95(3), 328–333. 10.1007/bf00320984 28314006

[jame21739-bib-0060] Sillman, S. (1999). The relation between ozone, NOx and hydrocarbons in urban and polluted rural environments. Atmospheric Environment, 33(12), 1821–1845. 10.1016/S1352-2310(98)00345-8

[jame21739-bib-0061] Stavrakou, T. , Müller, J. F. , Bauwens, M. , De Smedt, I. , Van Roozendael, M. , De Mazière, M. , et al. (2015). How consistent are top‐down hydrocarbon emissions based on formaldehyde observations from GOME‐2 and OMI? Atmospheric Chemistry and Physics, 15(20), 11861–11884. 10.5194/acp-15-11861-2015

[jame21739-bib-0062] Stavrakou, T. , Müller, J. F. , Bauwens, M. , Smedt, I. , Roozendael, M. , & Guenther, A. (2018). Impact of short‐term climate variability on volatile organic compounds emissions assessed using OMI satellite formaldehyde observations. Geophysical Research Letters, 0(16), 8681–8689. 10.1029/2018GL078676

[jame21739-bib-0063] Stavrakou, T. , Müller, J.‐F. , Smedt, I. D. , Roozendael, M. V. , Van der Werf, G. , Giglio, L. , & Guenther, A. (2009). Evaluating the performance of pyrogenic and biogenic emission inventories against one decade of space‐based formaldehyde columns. Atmospheric Chemistry and Physics, 9(3), 1037–1060. 10.5194/acp-9-1037-2009

[jame21739-bib-0064] Stull, R. B. (1988). An introduction to boundary layer meteorology. Springer Science & Business Media.

[jame21739-bib-0065] Tilmes, S. , Hodzic, A. , Emmons, L. K. , Mills, M. J. , Gettelman, A. , Kinnison, D. E. , et al. (2019). Climate forcing and trends of organic aerosols in the community Earth system model (CESM2). Journal of Advances in Modeling Earth Systems, 11(12), 4323–4351. 10.1029/2019MS001827

[jame21739-bib-0066] Tingey, D. T. , Evans, R. , & Gumpertz, M. (1981). Effects of environmental conditions on isoprene emission from live oak. Planta, 152(6), 565–570. 10.1007/BF00380829 24301162

[jame21739-bib-0067] Veefkind, J. P. , Aben, I. , McMullan, K. , Förster, H. , de Vries, J. , Otter, G. , et al. (2012). TROPOMI on the ESA sentinel‐5 precursor: A GMES mission for global observations of the atmospheric composition for climate, air quality and ozone layer applications. Remote Sensing of Environment, 120, 70–83. 10.1016/j.rse.2011.09.027

[jame21739-bib-0068] Wang, P. , Liu, Y. , Dai, J. , Fu, X. , Wang, X. , Guenther, A. , & Wang, T. (2021). Isoprene emissions response to drought and the impacts on ozone and SOA in China. Journal of Geophysical Research: Atmospheres, 126(10), e2020JD033263. 10.1029/2020JD033263

[jame21739-bib-0069] Wells, K. C. , Millet, D. B. , Payne, V. H. , Deventer, M. J. , Bates, K. H. , de Gouw, J. A. , et al. (2020). Satellite isoprene retrievals constrain emissions and atmospheric oxidation. Nature, 585(7824), 225–233. 10.1038/s41586-020-2664-3 32908268 PMC7490801

[jame21739-bib-0070] Wolfe, G. M. , Kaiser, J. , Hanisco, T. F. , Keutsch, F. N. , de Gouw, J. A. , Gilman, J. B. , et al. (2016). Formaldehyde production from isoprene oxidation across NOx regimes. Atmospheric Chemistry and Physics, 16(4), 2597–2610. 10.5194/acp-16-2597-2016 29619046 PMC5879783

[jame21739-bib-0071] Yan, H. , Wang, S.‐q. , Billesbach, D. , Oechel, W. , Bohrer, G. , Meyers, T. , et al. (2015). Improved global simulations of gross primary product based on a new definition of water stress factor and a separate treatment of C3 and C4 plants. Ecological Modelling, 297, 42–59. 10.1016/j.ecolmodel.2014.11.002

[jame21739-bib-0072] Zheng, Y. , Unger, N. , Tadić, J. M. , Seco, R. , Guenther, A. B. , Barkley, M. P. , et al. (2017). Drought impacts on photosynthesis, isoprene emission and atmospheric formaldehyde in a mid‐latitude forest. Atmospheric Environment, 167, 190–201. 10.1016/j.atmosenv.2017.08.017

[jame21739-bib-0073] Zhu, L. , Mickley, L. J. , Jacob, D. J. , Marais, E. A. , Sheng, J. , Hu, L. , et al. (2017). Long‐term (2005–2014) trends in formaldehyde (HCHO) columns across North America as seen by the OMI satellite instrument: Evidence of changing emissions of volatile organic compounds. Geophysical Research Letters, 44(13), 7079–7086. 10.1002/2017GL073859

