# Supplementary material for: Modeling Isoprene Emission Response to Drought and Heatwaves Within MEGAN Using Evapotranspiration Data and by Coupling With the Community Land Model
Source: J Adv Model Earth Syst. 2022 Dec 21;14(12):e2022MS003174. doi: 10.1029/2022MS003174 (PMC10078486; doi:10.1029/2022MS003174)
Supplement: Supplementary file 1 — Supporting Information S1 [file JAME-14-0-s001.docx]

**Supplement of “Modeling isoprene emission response to drought and heatwaves within MEGAN using evapotranspiration data and by coupling with the Community Land Model”**

Hui Wang^1*^, Xinchen Lu^2^, Roger Seco^3^, Trissevgeni Stavrakou^4^, Thomas Karl^5^, Xiaoyan Jiang^1^, Lianhong Gu^6^, Alex B. Guenther^1*^

1. Department of Earth System Science, University of California, Irvine, California, USA

2. Department of Environmental Science, Policy and Management, University of California, Berkeley, California, USA

3. Institute of Environmental Assessment and Water Research (IDAEA-CSIC), Barcelona, Catalonia, Spain

4. Royal Belgian Royal Institute for Space Aeronomy, Brussels, Belgium

5. Department of Atmospheric and Cryospheric Sciences, University of Innsbruck, Innsbruck, Austria

6. Environmental Sciences Division and Climate Change Science Institute, Oak Ridge National Laboratory, Oak Ridge, Tennessee, USA

Correspondence to: Hui Wang (huiw16@uci.edu) & Alex B. Guenther (aguenthe@uci.edu)


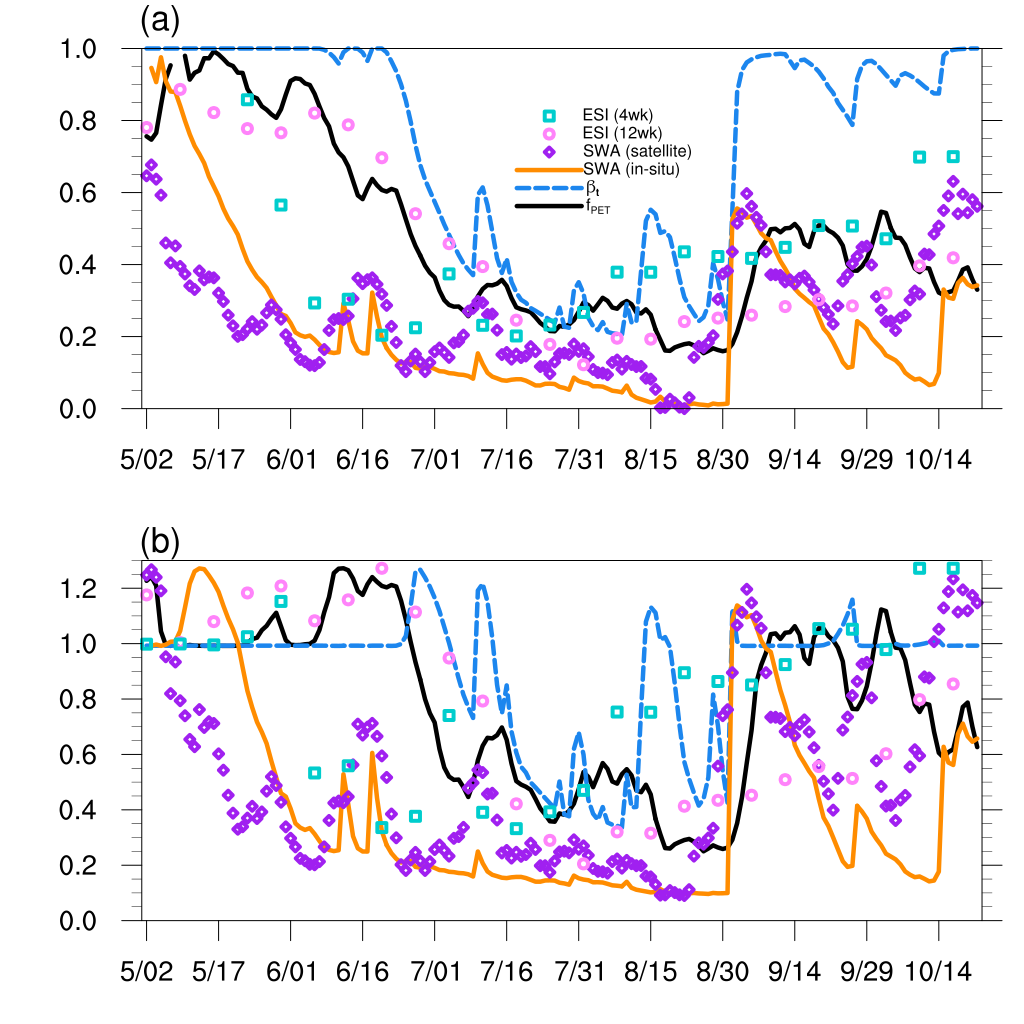


Figure S1. Comparison of the different normalized drought indexes including ESI drought indexes over 4-week (cyan square) and 12-week (pink circle) periods, the Soil Water Availability (SWA) from the in-situ (purple diamond) and satellite (orange solid line) observations, βt (blue dashed line) from the CLM model and the 7 day running averaged normalized f_PET_ (black solid line) in this study. The outputs of the Parameterized Drought Stress (PDS) offline algorithm with above inputs are presented in (b).


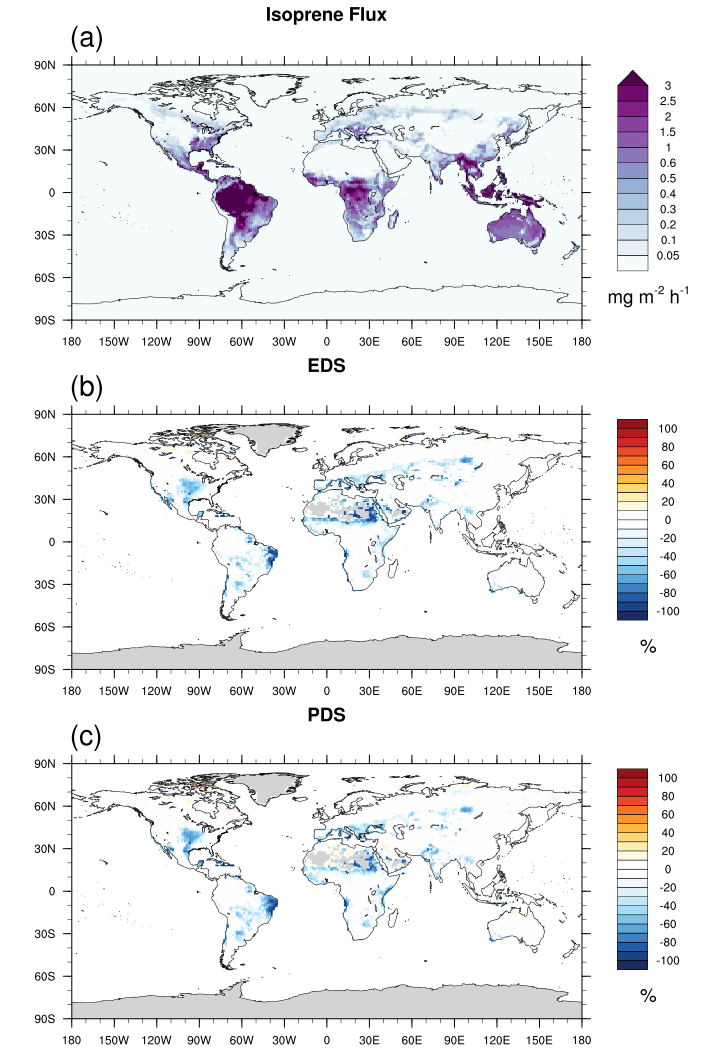


Figure S2. The spatial distribution of the isoprene flux estimated by CLM in 2012 (b) and the impact of drought estimated by the EDS algorithm (b) and the PDS algorithm (c).


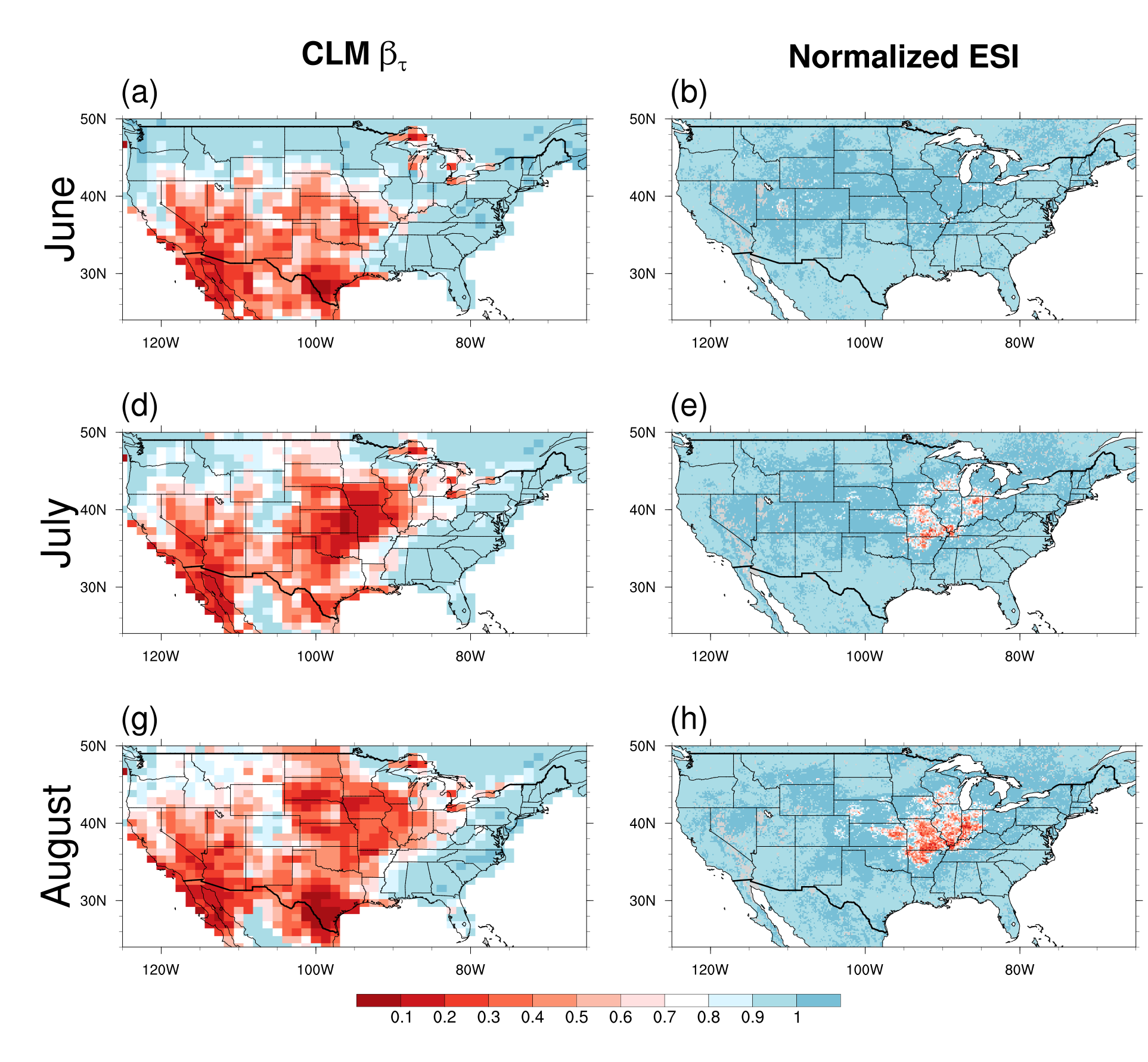


Figure S3. The spatial distributions of β_t_ simulated by CLM5 and the normalized satellite evaporative stress index (ESI) during the summer. The three rows represent different months from June to August.


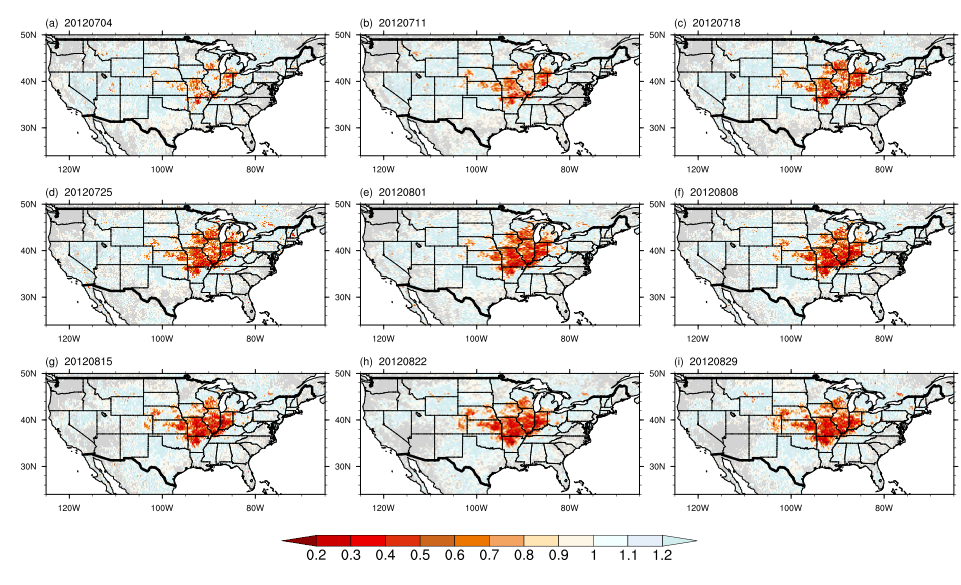


Figure S4. The spatial distributions of γ_sm_ calculated by the offline Parameterized Drought Stress (PDS) algorithm in July-August 2012 using the satellite Evapotranspiration Stress Index (ESI) inputs.


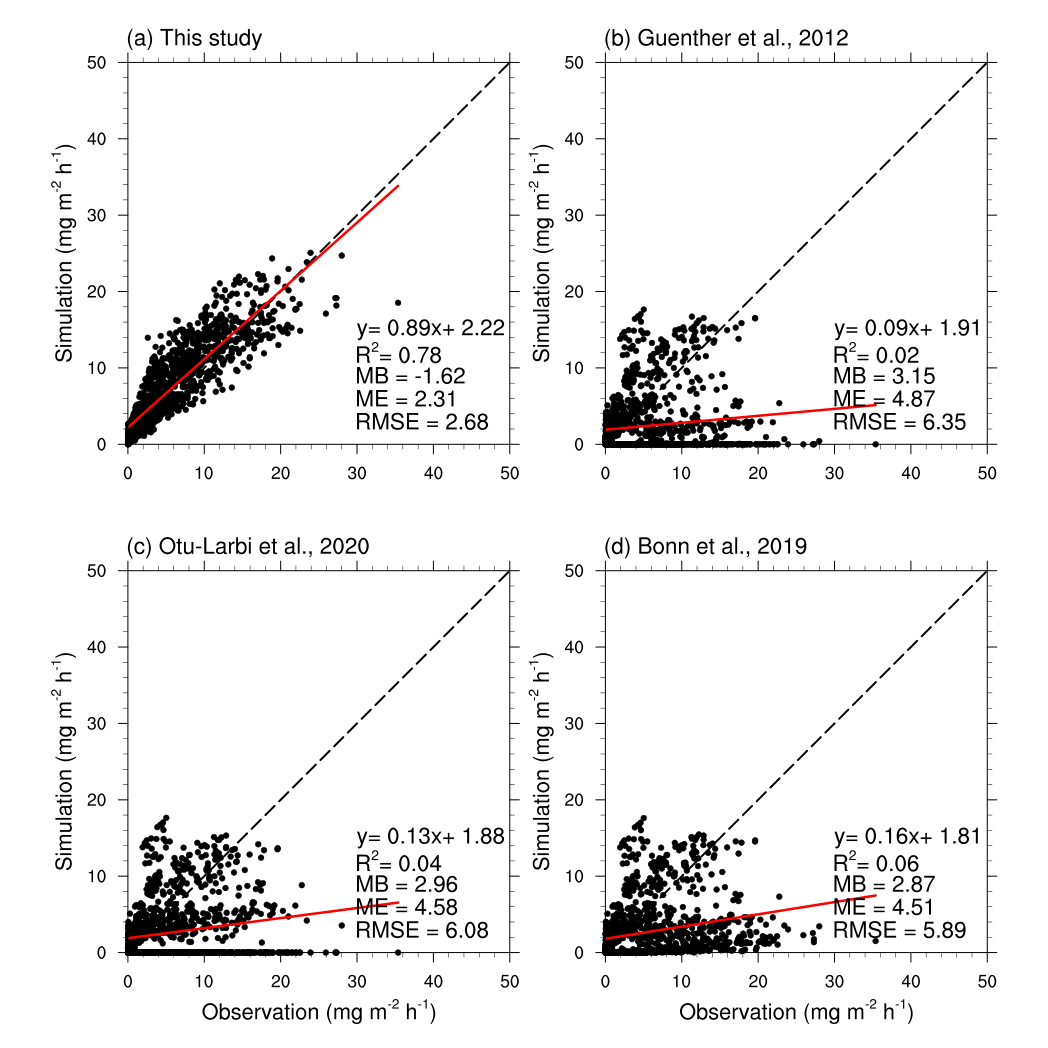


Figure S5. Scatter plots of measured diurnal isoprene fluxes and modelled diurnal isoprene fluxes with different drought algorithms (see Table 2) and the wilting point of 0.23 m^3^ m^-3^.
